# Supplementary figures and images for: Breaking antimicrobial resistance by disrupting extracytoplasmic protein folding
Source: eLife. 2022 Jan 13;11:e57974. doi: 10.7554/eLife.57974 (PMC8863373; doi:10.7554/eLife.57974)

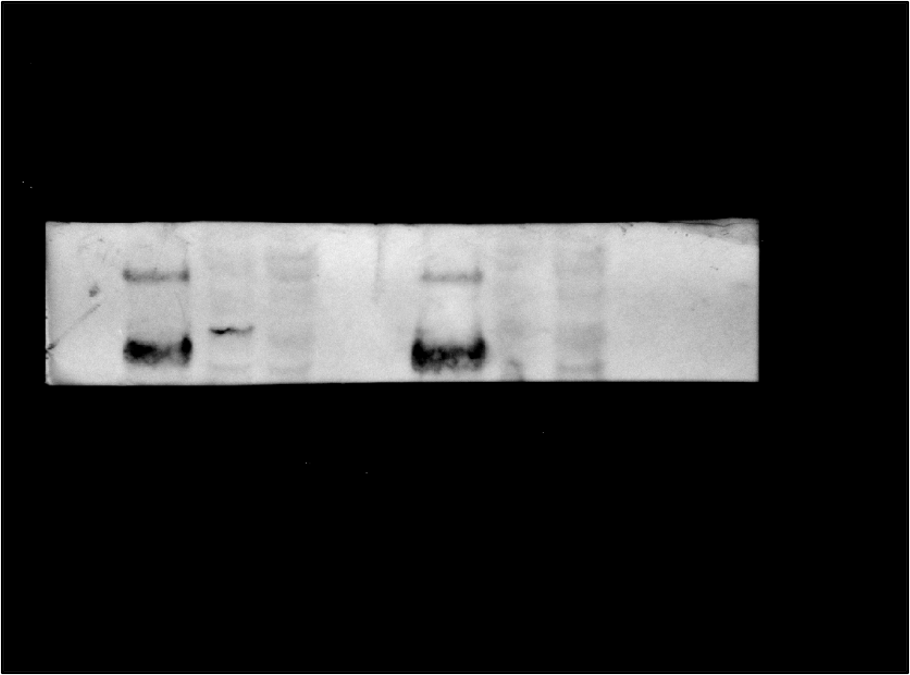

Supplement: Figure 2—source data 1. — ‘Top Panel’ in the file name refers to immunoblots carried out using a Strep-Tactin-AP conjugate, while ‘Bottom Panel’ refers to immunoblots carried out using an anti-DnaK 8E2/2 antibody. ‘Left’ and ‘Right’ in the file names refer to the part of the immunoblot to the left or to the right of the vertical black line shown in the final figure, respectively. [file elife-57974-fig2-data1.zip › Figure 2-source data 1/TopPanel_Right.tif]

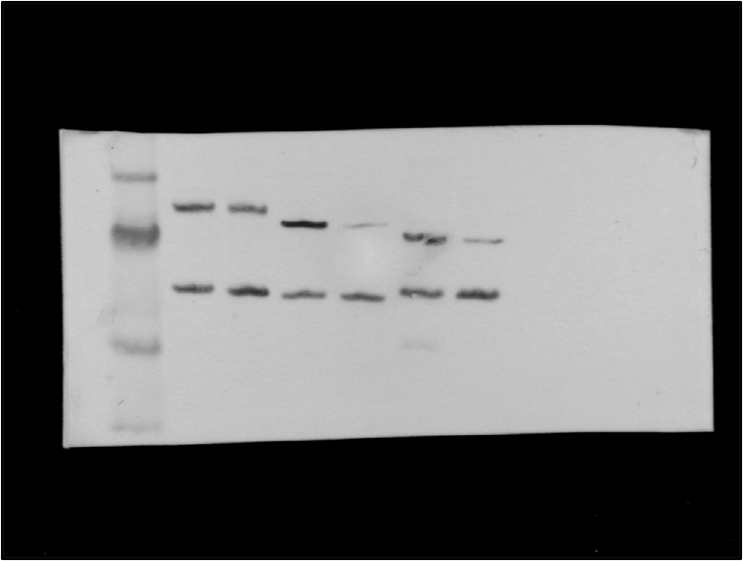

Supplement: Figure 2—source data 1. — ‘Top Panel’ in the file name refers to immunoblots carried out using a Strep-Tactin-AP conjugate, while ‘Bottom Panel’ refers to immunoblots carried out using an anti-DnaK 8E2/2 antibody. ‘Left’ and ‘Right’ in the file names refer to the part of the immunoblot to the left or to the right of the vertical black line shown in the final figure, respectively. [file elife-57974-fig2-data1.zip › Figure 2-source data 1/TopPanel_Left.tif]

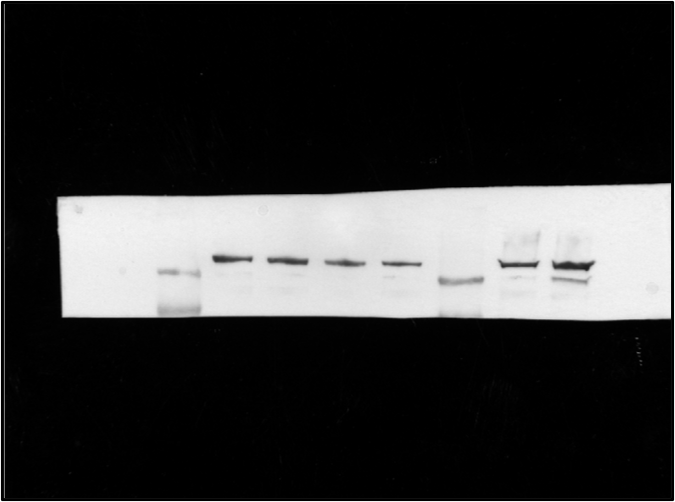

Supplement: Figure 2—source data 1. — ‘Top Panel’ in the file name refers to immunoblots carried out using a Strep-Tactin-AP conjugate, while ‘Bottom Panel’ refers to immunoblots carried out using an anti-DnaK 8E2/2 antibody. ‘Left’ and ‘Right’ in the file names refer to the part of the immunoblot to the left or to the right of the vertical black line shown in the final figure, respectively. [file elife-57974-fig2-data1.zip › Figure 2-source data 1/BottomPanel_Righ.tif]

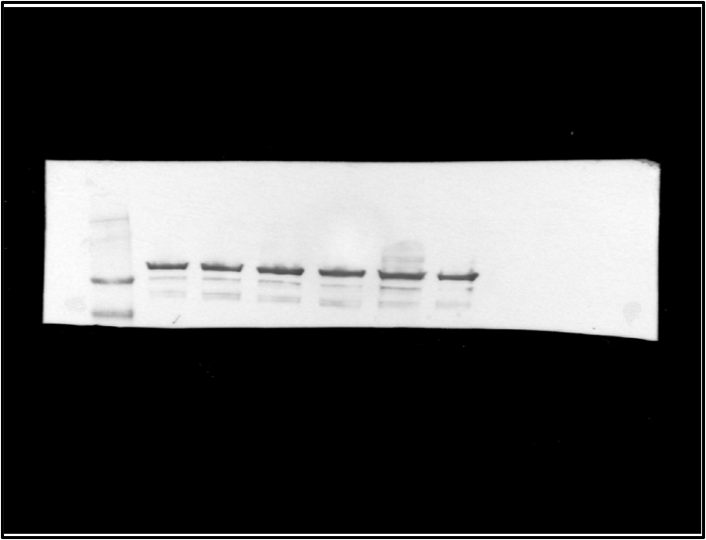

Supplement: Figure 2—source data 1. — ‘Top Panel’ in the file name refers to immunoblots carried out using a Strep-Tactin-AP conjugate, while ‘Bottom Panel’ refers to immunoblots carried out using an anti-DnaK 8E2/2 antibody. ‘Left’ and ‘Right’ in the file names refer to the part of the immunoblot to the left or to the right of the vertical black line shown in the final figure, respectively. [file elife-57974-fig2-data1.zip › Figure 2-source data 1/BottomPanel_Left.tif]

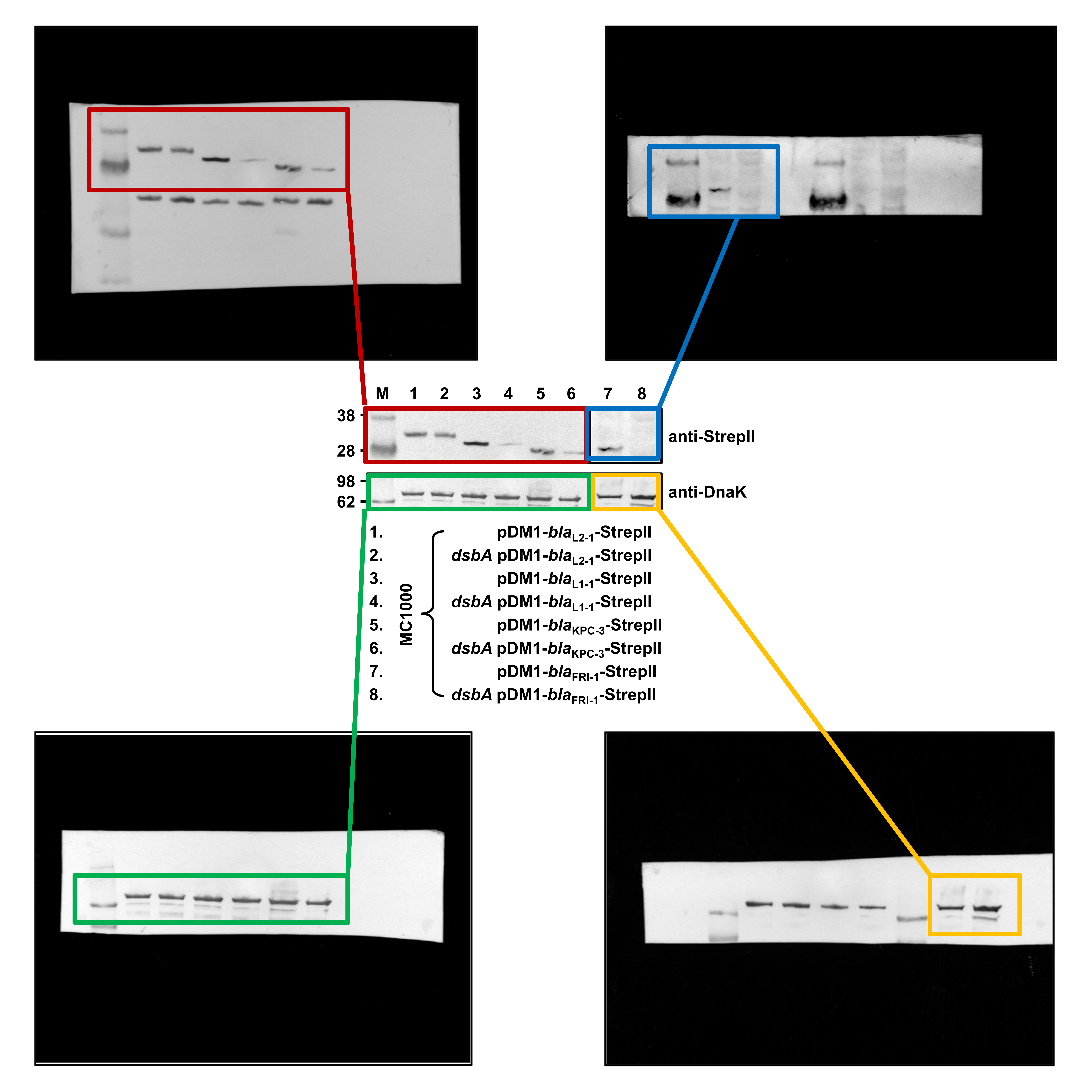

Supplement: Figure 2—source data 2. — The figure included in the paper is shown in the center and relevant bands used for each part of the figure are marked with color-coded boxes on the uncropped immunoblots. [file elife-57974-fig2-data2.zip › Figure 2-source data 2/Figure 2A.tif]

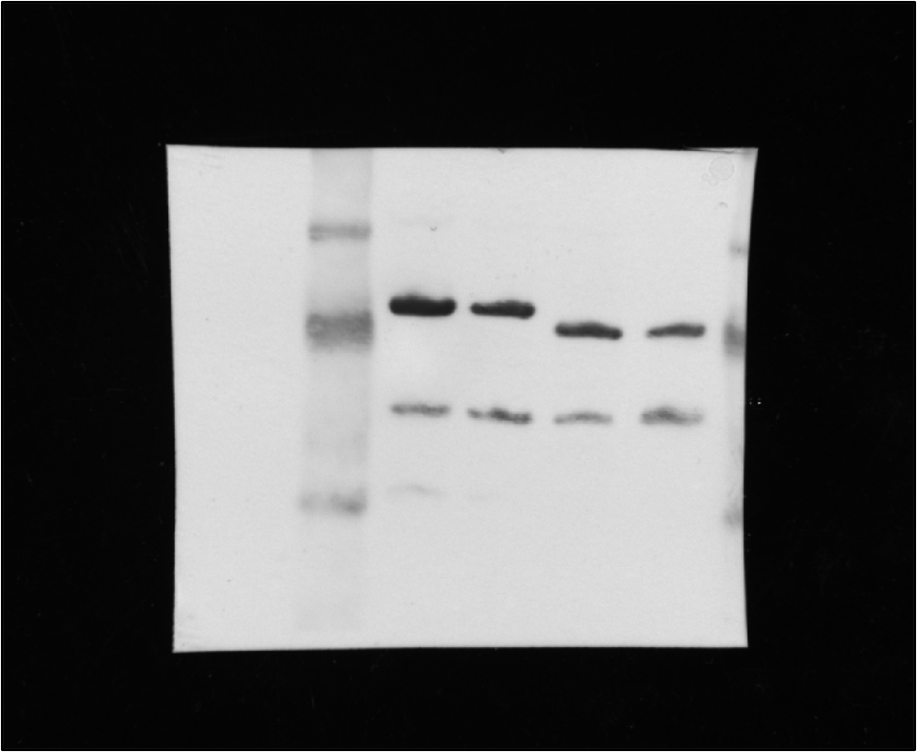

Supplement: Figure 2—source data 3. — ‘Top Panel’ in the file name refers to immunoblots carried out using a Strep-Tactin-AP conjugate or a Strep-Tactin-HRP conjugate, while ‘Bottom Panel’ refers to immunoblots carried out using an anti-DnaK 8E2/2 antibody. ‘Left’, ‘Middle’, and ‘Right’ in the file names refer to the part of the immunoblot to the left, in-between, or to the right of the vertical black lines shown in the final figure, respectively. [file elife-57974-fig2-data3.zip › Figure 2-source data 3/TopPanel_Right.tif]

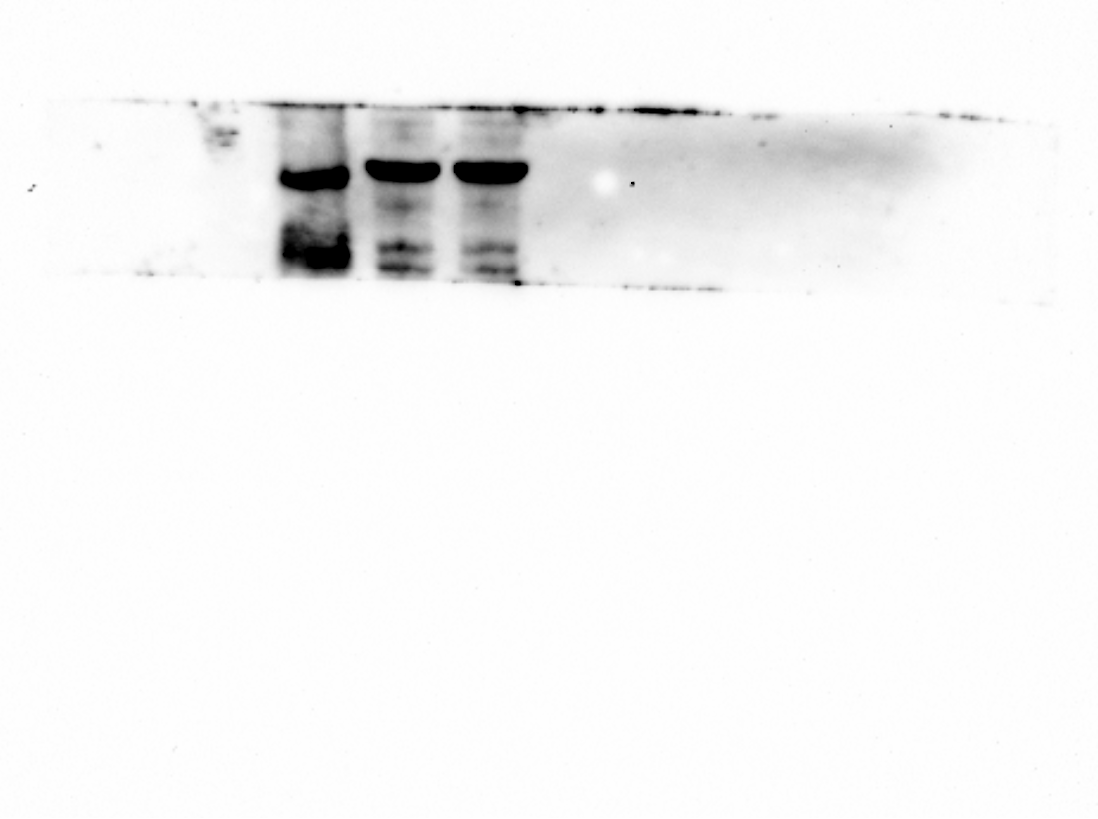

Supplement: Figure 2—source data 3. — ‘Top Panel’ in the file name refers to immunoblots carried out using a Strep-Tactin-AP conjugate or a Strep-Tactin-HRP conjugate, while ‘Bottom Panel’ refers to immunoblots carried out using an anti-DnaK 8E2/2 antibody. ‘Left’, ‘Middle’, and ‘Right’ in the file names refer to the part of the immunoblot to the left, in-between, or to the right of the vertical black lines shown in the final figure, respectively. [file elife-57974-fig2-data3.zip › Figure 2-source data 3/TopPanel_Middle.tif]

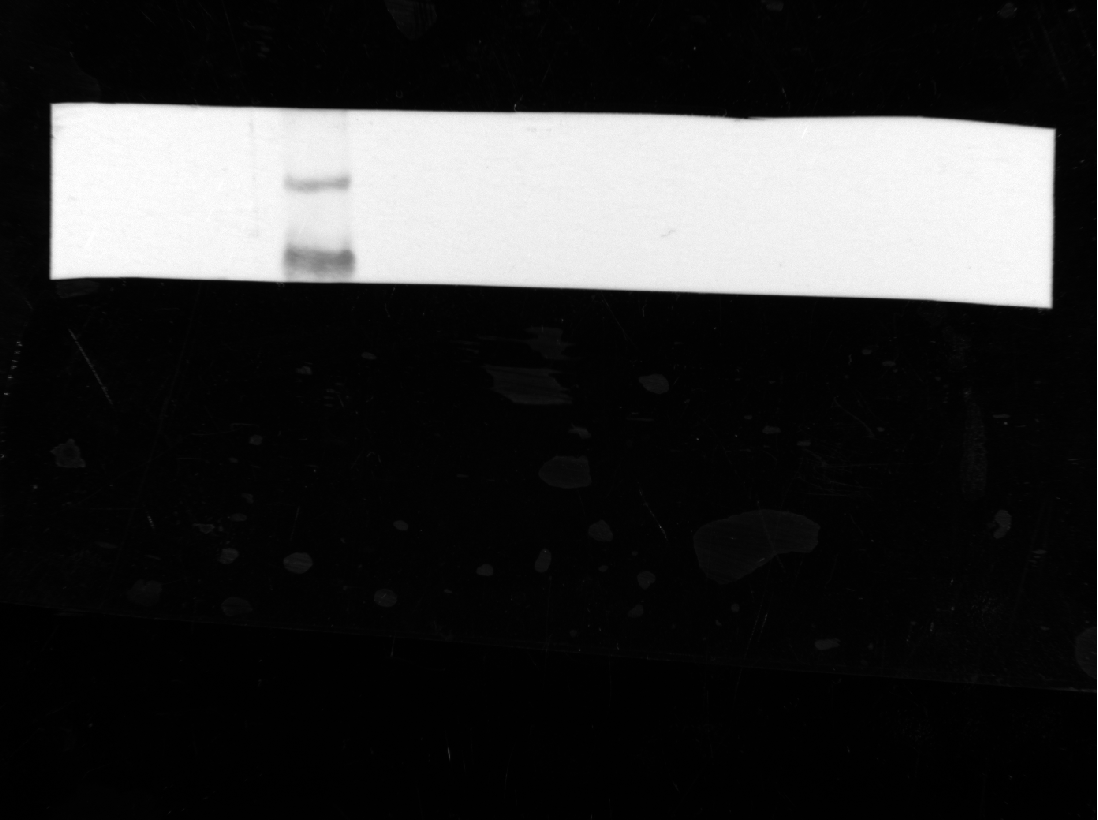

Supplement: Figure 2—source data 3. — ‘Top Panel’ in the file name refers to immunoblots carried out using a Strep-Tactin-AP conjugate or a Strep-Tactin-HRP conjugate, while ‘Bottom Panel’ refers to immunoblots carried out using an anti-DnaK 8E2/2 antibody. ‘Left’, ‘Middle’, and ‘Right’ in the file names refer to the part of the immunoblot to the left, in-between, or to the right of the vertical black lines shown in the final figure, respectively. [file elife-57974-fig2-data3.zip › Figure 2-source data 3/TopPanel_Left.tif]

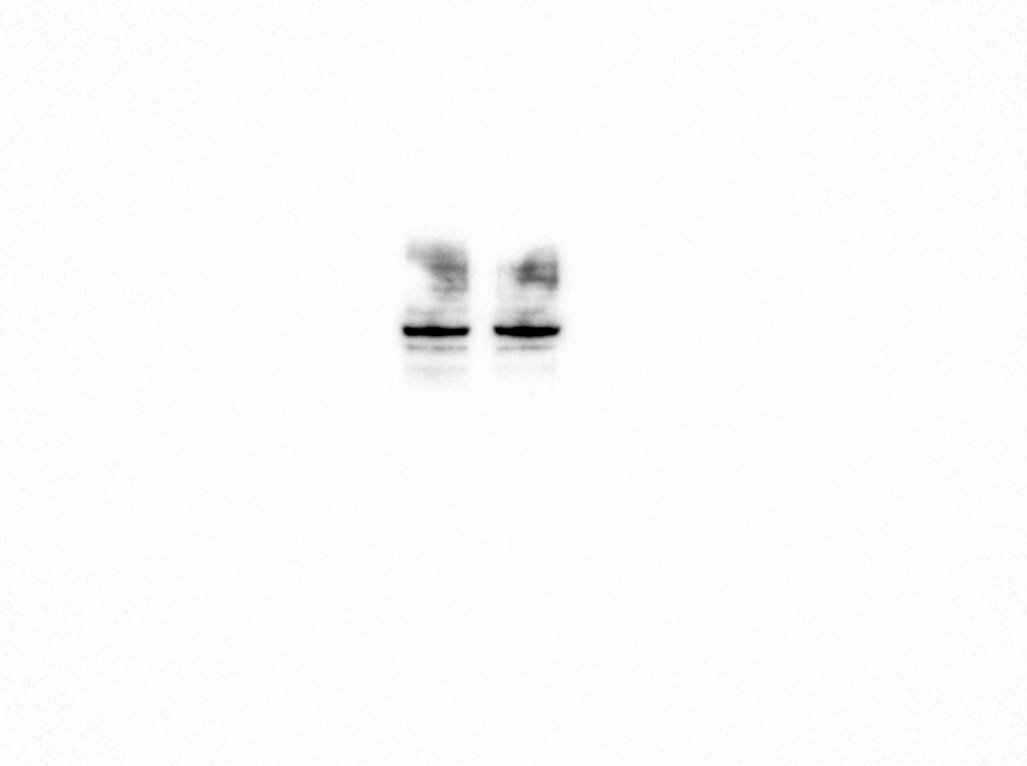

Supplement: Figure 2—source data 3. — ‘Top Panel’ in the file name refers to immunoblots carried out using a Strep-Tactin-AP conjugate or a Strep-Tactin-HRP conjugate, while ‘Bottom Panel’ refers to immunoblots carried out using an anti-DnaK 8E2/2 antibody. ‘Left’, ‘Middle’, and ‘Right’ in the file names refer to the part of the immunoblot to the left, in-between, or to the right of the vertical black lines shown in the final figure, respectively. [file elife-57974-fig2-data3.zip › Figure 2-source data 3/BottomPanel_Middle.tif]

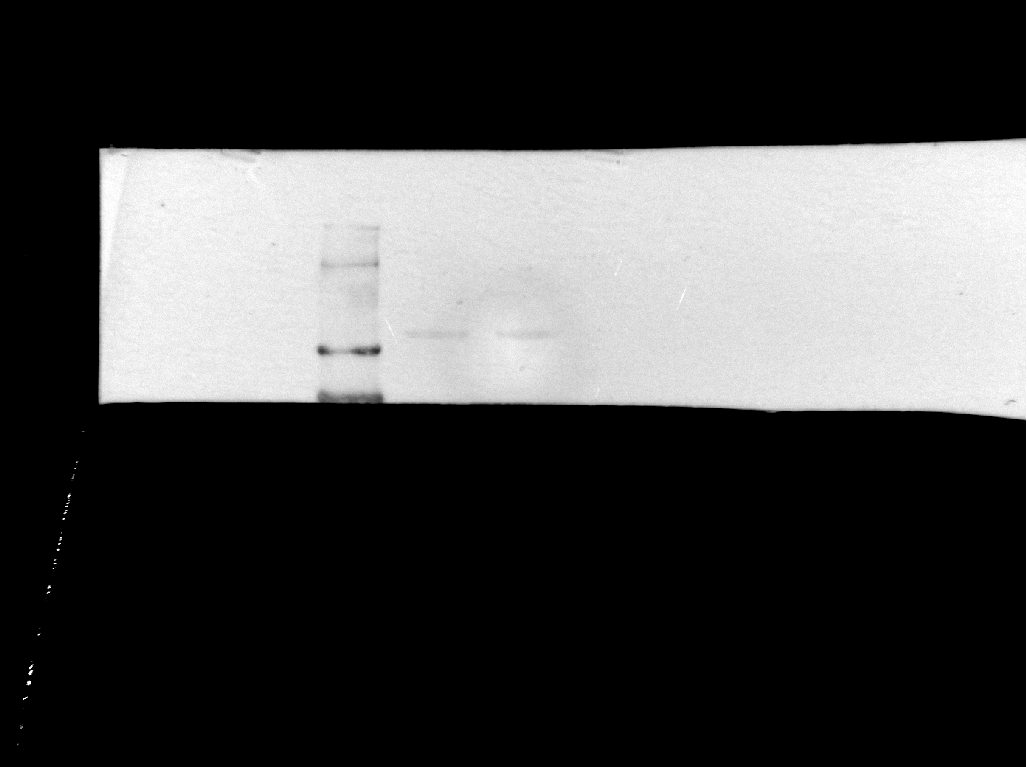

Supplement: Figure 2—source data 3. — ‘Top Panel’ in the file name refers to immunoblots carried out using a Strep-Tactin-AP conjugate or a Strep-Tactin-HRP conjugate, while ‘Bottom Panel’ refers to immunoblots carried out using an anti-DnaK 8E2/2 antibody. ‘Left’, ‘Middle’, and ‘Right’ in the file names refer to the part of the immunoblot to the left, in-between, or to the right of the vertical black lines shown in the final figure, respectively. [file elife-57974-fig2-data3.zip › Figure 2-source data 3/BottomPanel_Left.tif]

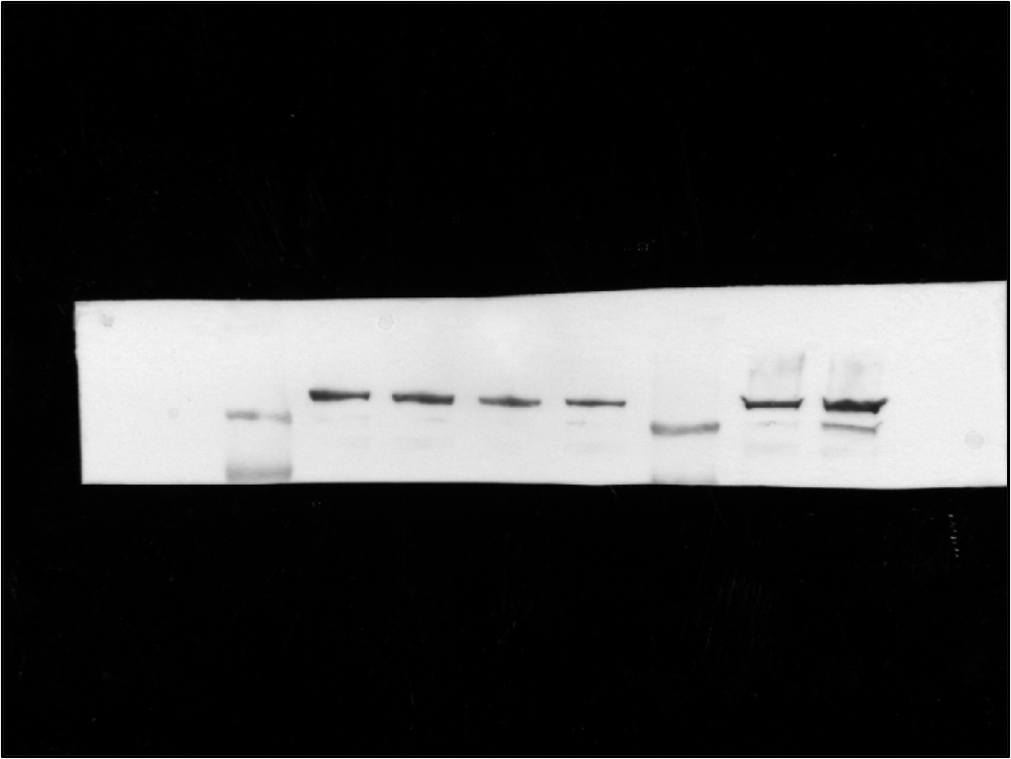

Supplement: Figure 2—source data 3. — ‘Top Panel’ in the file name refers to immunoblots carried out using a Strep-Tactin-AP conjugate or a Strep-Tactin-HRP conjugate, while ‘Bottom Panel’ refers to immunoblots carried out using an anti-DnaK 8E2/2 antibody. ‘Left’, ‘Middle’, and ‘Right’ in the file names refer to the part of the immunoblot to the left, in-between, or to the right of the vertical black lines shown in the final figure, respectively. [file elife-57974-fig2-data3.zip › Figure 2-source data 3/BotomPanel_Right.tif]

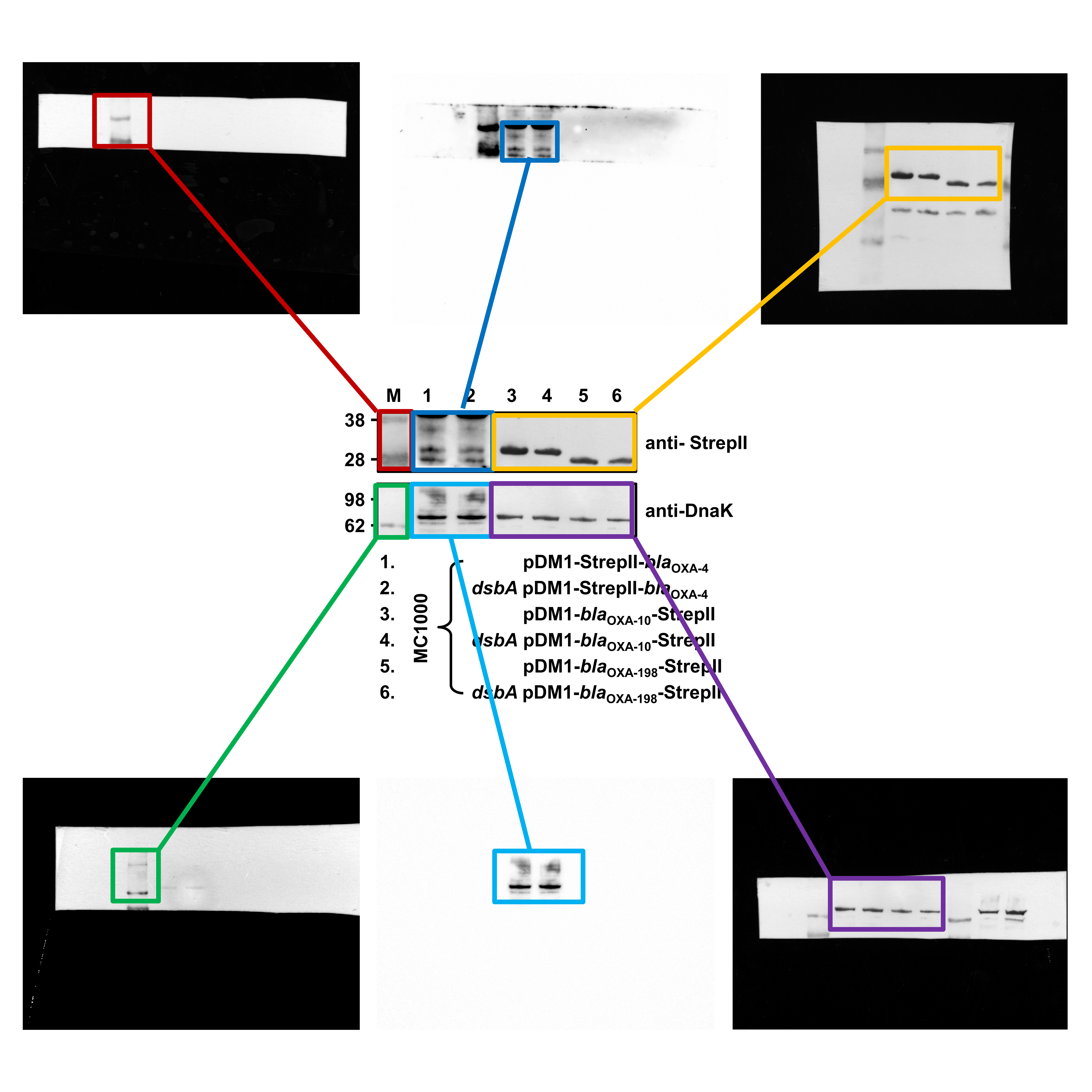

Supplement: Figure 2—source data 4. — The figure included in the paper is shown in the center and relevant bands used for each part of the figure are marked with color-coded boxes on the uncropped immunoblots. [file elife-57974-fig2-data4.zip › Figure 2-source data 4/Figure 2B.tif]

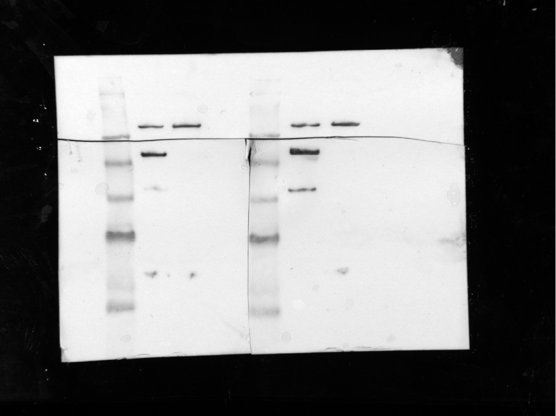

Supplement: Figure 3—source data 1. — The file names indicate the lanes of the immunoblot included in the paper that each of these files corresponds to. [file elife-57974-fig3-data1.zip › Figure 3-source data 1/LanesM-2_and_7-8.tif]

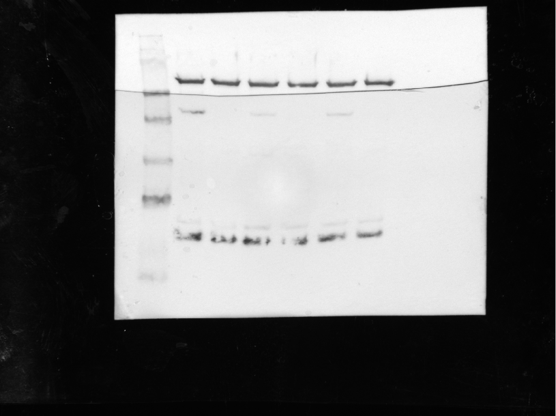

Supplement: Figure 3—source data 1. — The file names indicate the lanes of the immunoblot included in the paper that each of these files corresponds to. [file elife-57974-fig3-data1.zip › Figure 3-source data 1/Lanes3-6_and_9-8.tif]

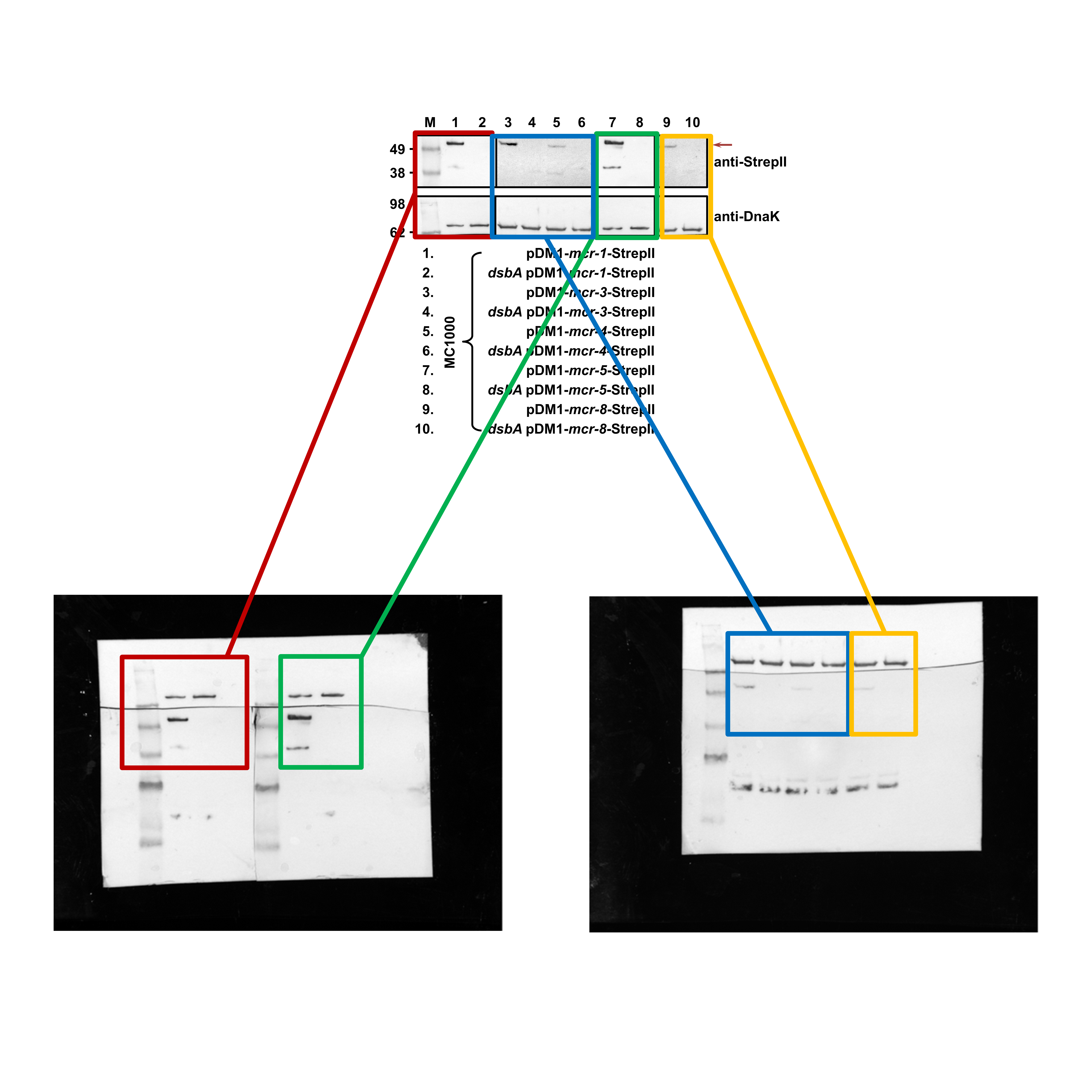

Supplement: Figure 3—source data 2. — The figure included in the paper is shown at the top and relevant bands used for each part of the figure are marked with color-coded boxes on the uncropped immunoblots. [file elife-57974-fig3-data2.zip › Figure 3-source data 2/Figure 3A.tif]

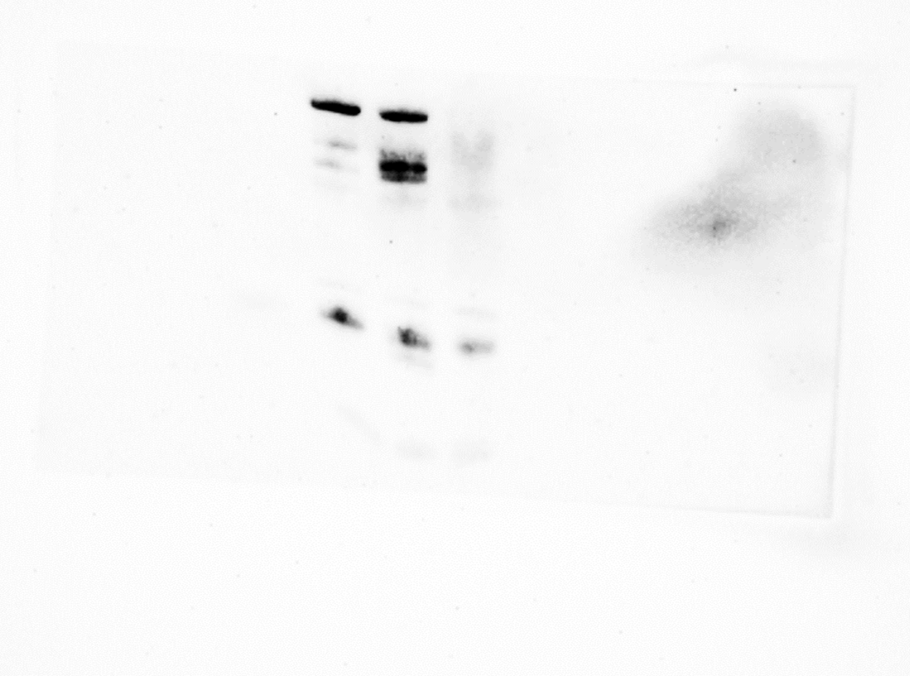

Supplement: Figure 4—source data 1. — ‘Top Panel’ in the file name refers to immunoblots carried out using an anti-HtrA1 (DegP) antibody, while ‘Bottom Panel’ refers to immunoblots carried out using an anti-DnaK 8E2/2 antibody. ‘Left’ and ‘Right’ in the file names refer to the part of the immunoblot to the left or to the right of the vertical black line shown in the final figure, respectively. [file elife-57974-fig4-data1.zip › Figure 4-source data 1/TopPanel_Right.tif]

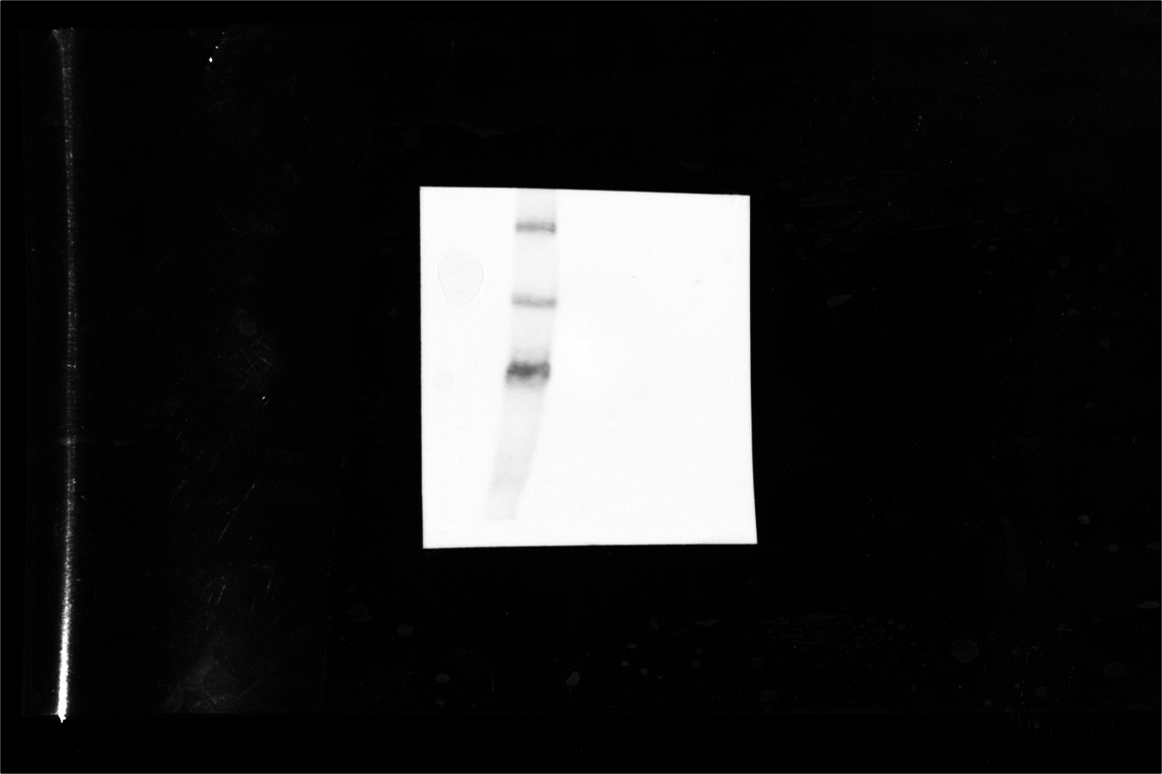

Supplement: Figure 4—source data 1. — ‘Top Panel’ in the file name refers to immunoblots carried out using an anti-HtrA1 (DegP) antibody, while ‘Bottom Panel’ refers to immunoblots carried out using an anti-DnaK 8E2/2 antibody. ‘Left’ and ‘Right’ in the file names refer to the part of the immunoblot to the left or to the right of the vertical black line shown in the final figure, respectively. [file elife-57974-fig4-data1.zip › Figure 4-source data 1/TopPanel_Left.tif]

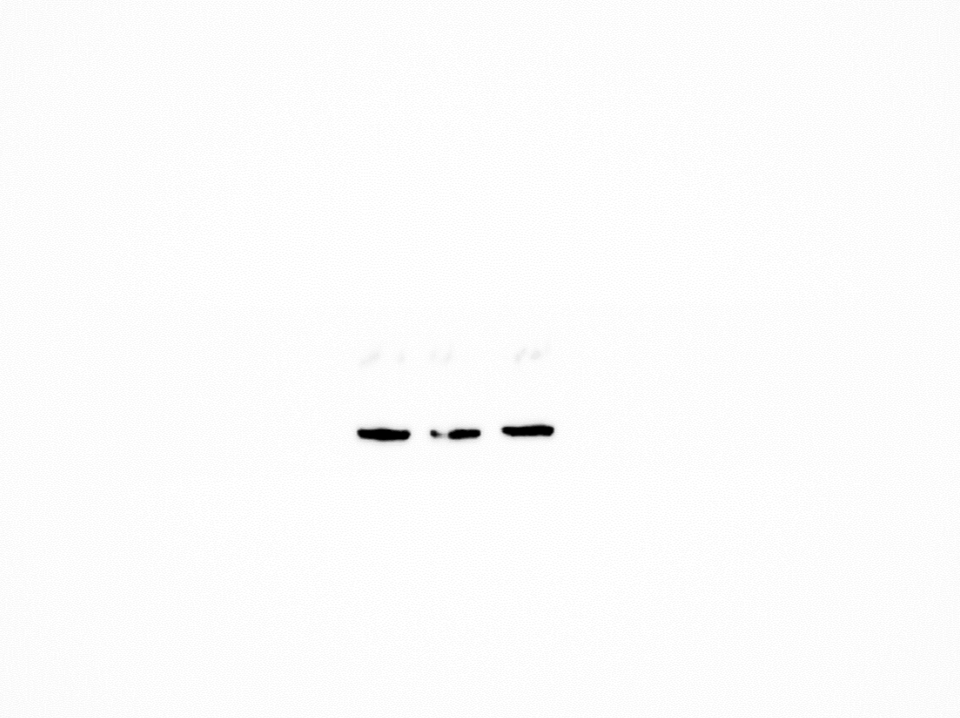

Supplement: Figure 4—source data 1. — ‘Top Panel’ in the file name refers to immunoblots carried out using an anti-HtrA1 (DegP) antibody, while ‘Bottom Panel’ refers to immunoblots carried out using an anti-DnaK 8E2/2 antibody. ‘Left’ and ‘Right’ in the file names refer to the part of the immunoblot to the left or to the right of the vertical black line shown in the final figure, respectively. [file elife-57974-fig4-data1.zip › Figure 4-source data 1/BottomPanel_Right.tif]

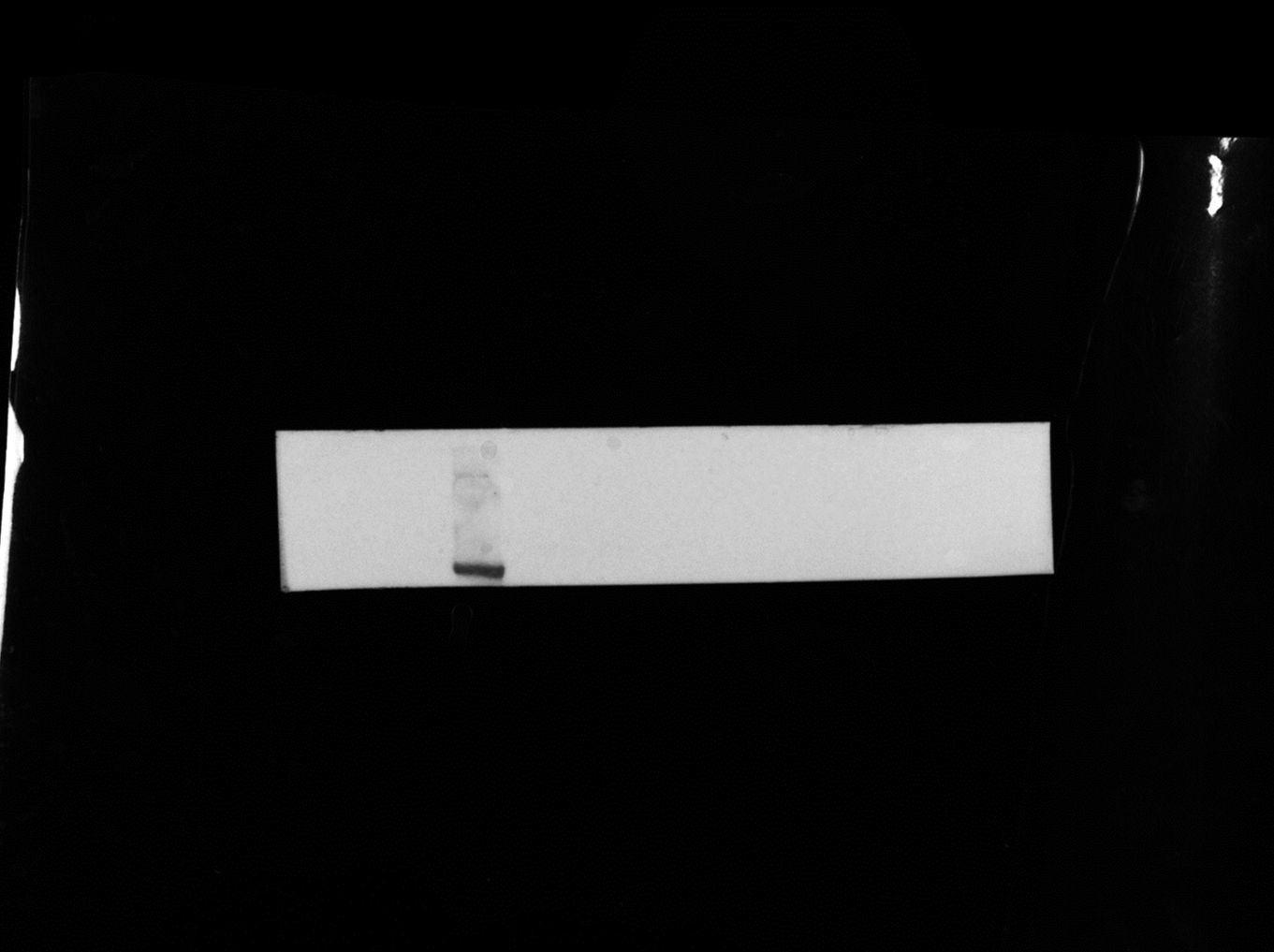

Supplement: Figure 4—source data 1. — ‘Top Panel’ in the file name refers to immunoblots carried out using an anti-HtrA1 (DegP) antibody, while ‘Bottom Panel’ refers to immunoblots carried out using an anti-DnaK 8E2/2 antibody. ‘Left’ and ‘Right’ in the file names refer to the part of the immunoblot to the left or to the right of the vertical black line shown in the final figure, respectively. [file elife-57974-fig4-data1.zip › Figure 4-source data 1/BottomPanel_Left.tif]

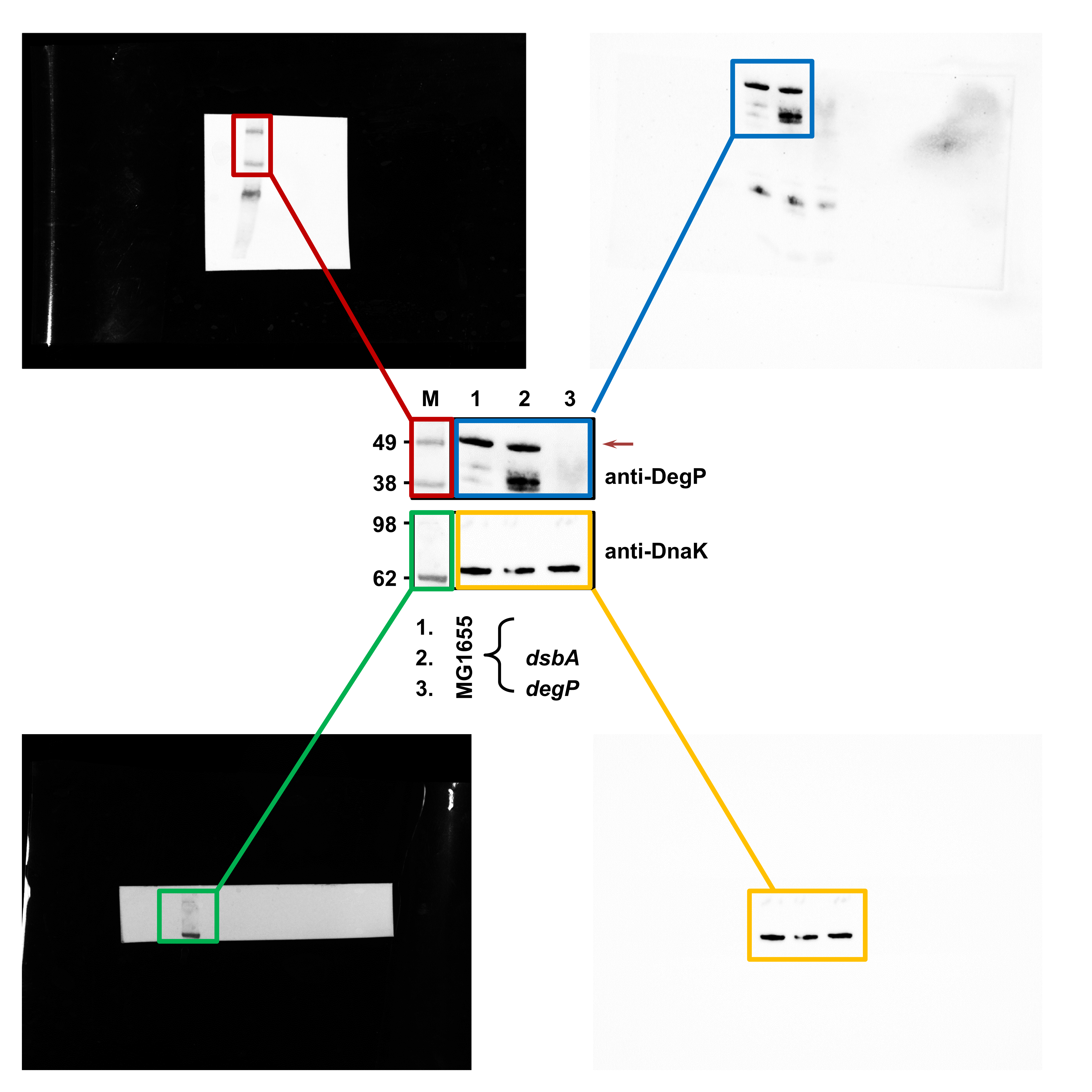

Supplement: Figure 4—source data 2. — The figure included in the paper is shown in the center and relevant bands used for each part of the figure are marked with color-coded boxes on the uncropped immunoblots. [file elife-57974-fig4-data2.zip › Figure 4-source data 2/Figure 4A.tif]

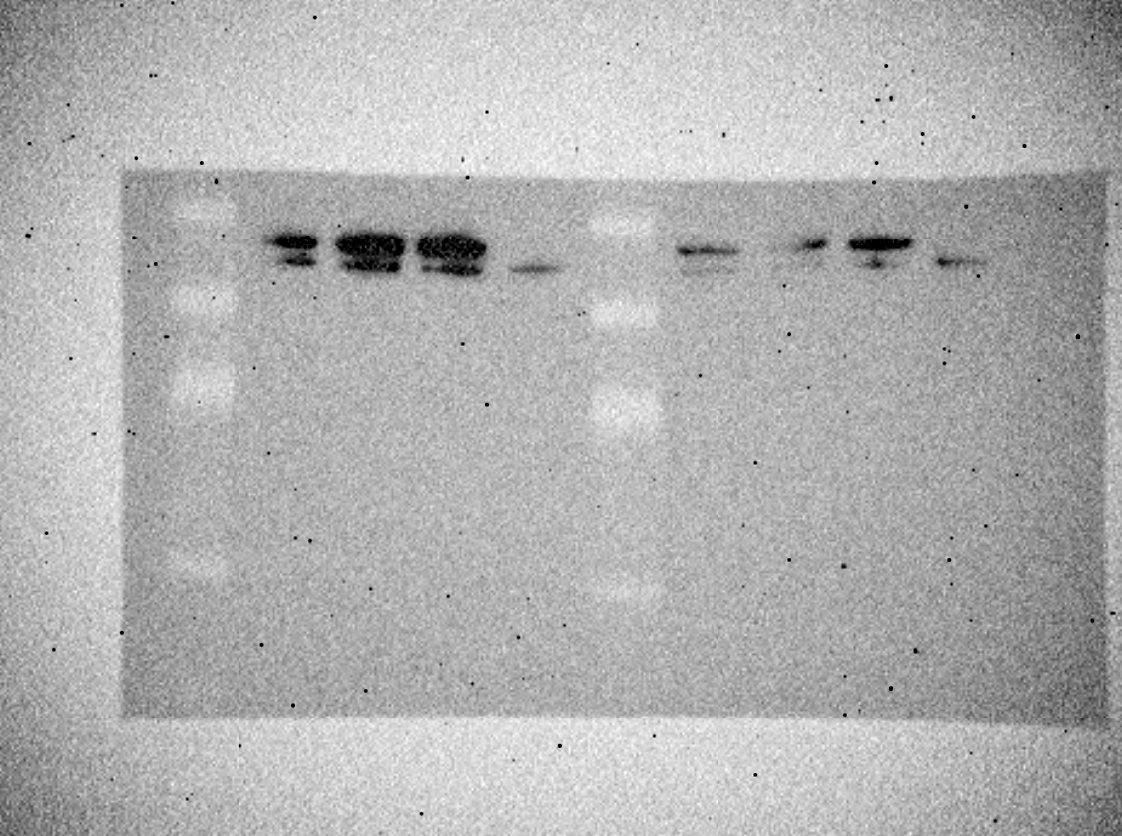

Supplement: Figure 4—source data 3. — ‘Top Panel’ in the file name refers to immunoblots carried out using an anti-AcrA antibody, while ‘Bottom Panel’ refers to immunoblots carried out using an anti-DnaK 8E2/2 antibody. ‘Left’ and ‘Right’ in the file names refer to the part of the immunoblot to the left or to the right of the vertical black line shown in the final figure, respectively. [file elife-57974-fig4-data3.zip › Figure 4-source data 3/TopPanel_Right.tif]

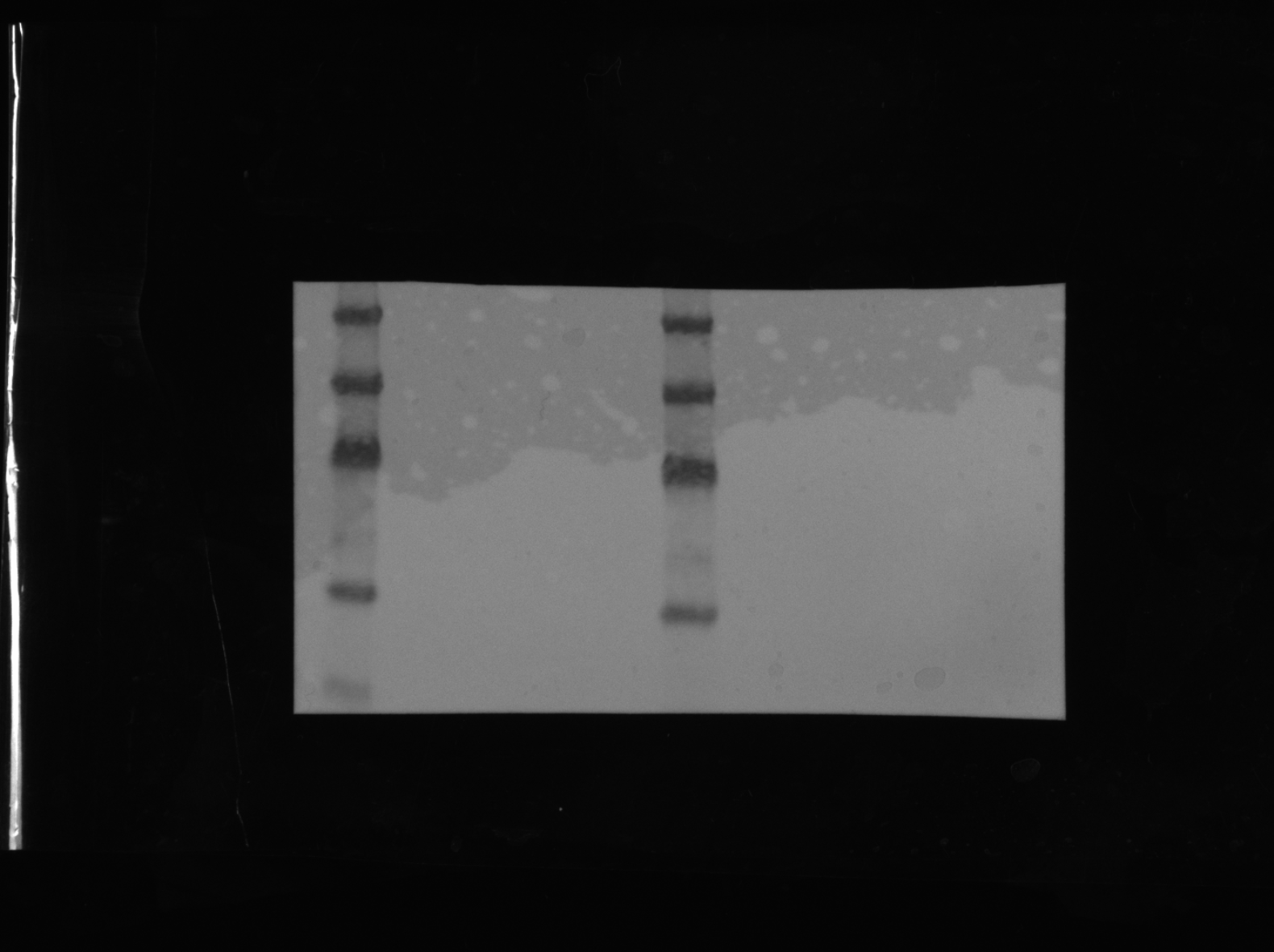

Supplement: Figure 4—source data 3. — ‘Top Panel’ in the file name refers to immunoblots carried out using an anti-AcrA antibody, while ‘Bottom Panel’ refers to immunoblots carried out using an anti-DnaK 8E2/2 antibody. ‘Left’ and ‘Right’ in the file names refer to the part of the immunoblot to the left or to the right of the vertical black line shown in the final figure, respectively. [file elife-57974-fig4-data3.zip › Figure 4-source data 3/TopPanel_Left.tif]

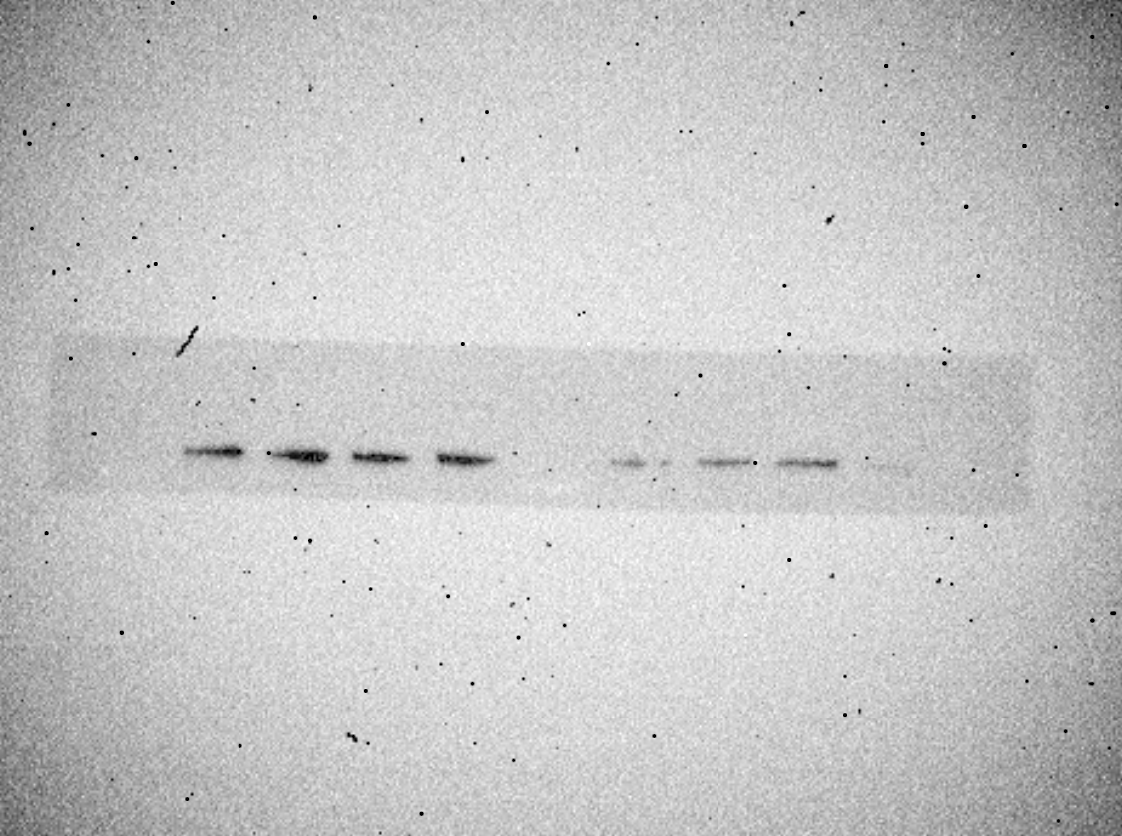

Supplement: Figure 4—source data 3. — ‘Top Panel’ in the file name refers to immunoblots carried out using an anti-AcrA antibody, while ‘Bottom Panel’ refers to immunoblots carried out using an anti-DnaK 8E2/2 antibody. ‘Left’ and ‘Right’ in the file names refer to the part of the immunoblot to the left or to the right of the vertical black line shown in the final figure, respectively. [file elife-57974-fig4-data3.zip › Figure 4-source data 3/BottomPanel_Right.tif]

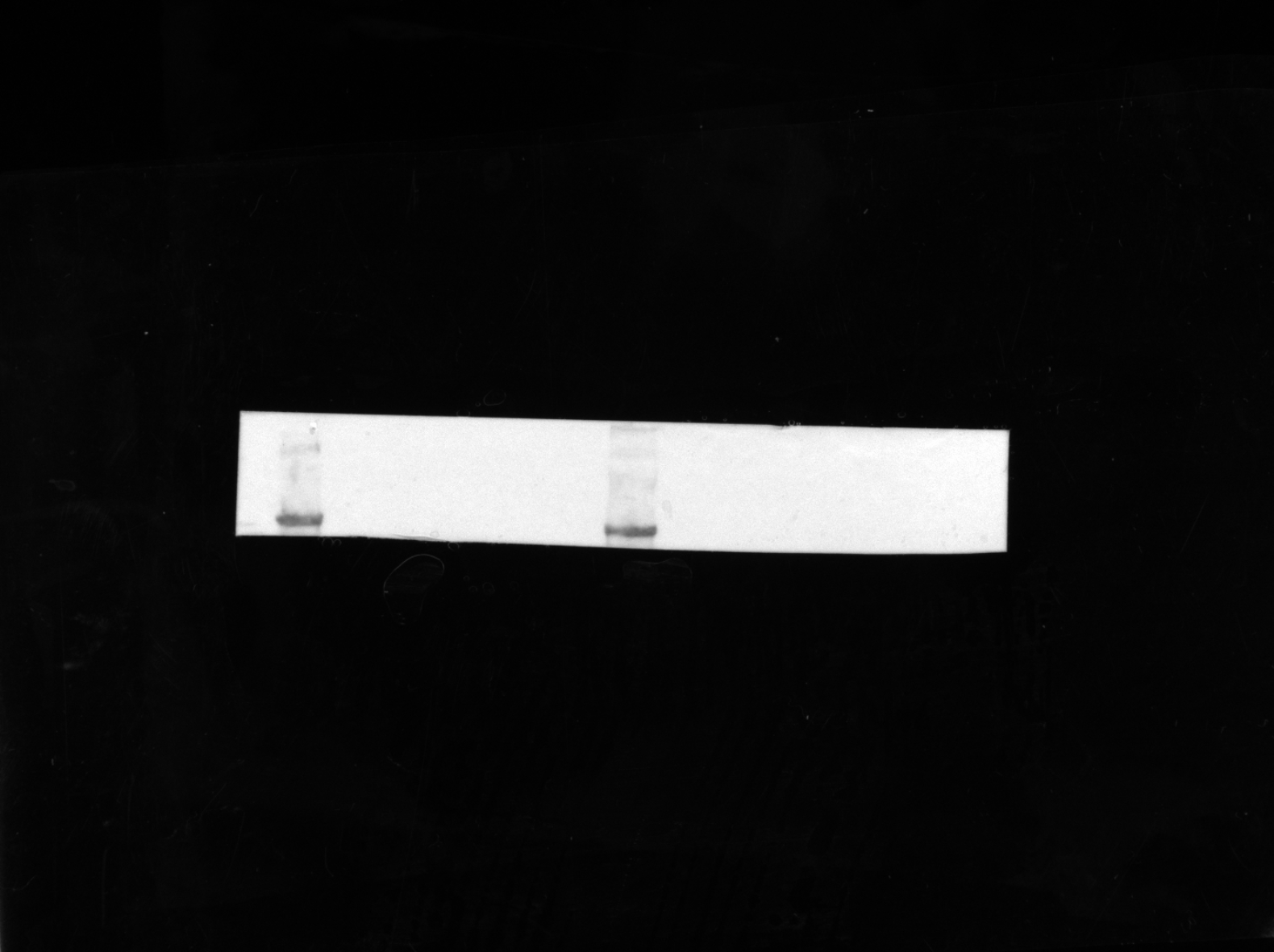

Supplement: Figure 4—source data 3. — ‘Top Panel’ in the file name refers to immunoblots carried out using an anti-AcrA antibody, while ‘Bottom Panel’ refers to immunoblots carried out using an anti-DnaK 8E2/2 antibody. ‘Left’ and ‘Right’ in the file names refer to the part of the immunoblot to the left or to the right of the vertical black line shown in the final figure, respectively. [file elife-57974-fig4-data3.zip › Figure 4-source data 3/BottomPanel_Left.tif]

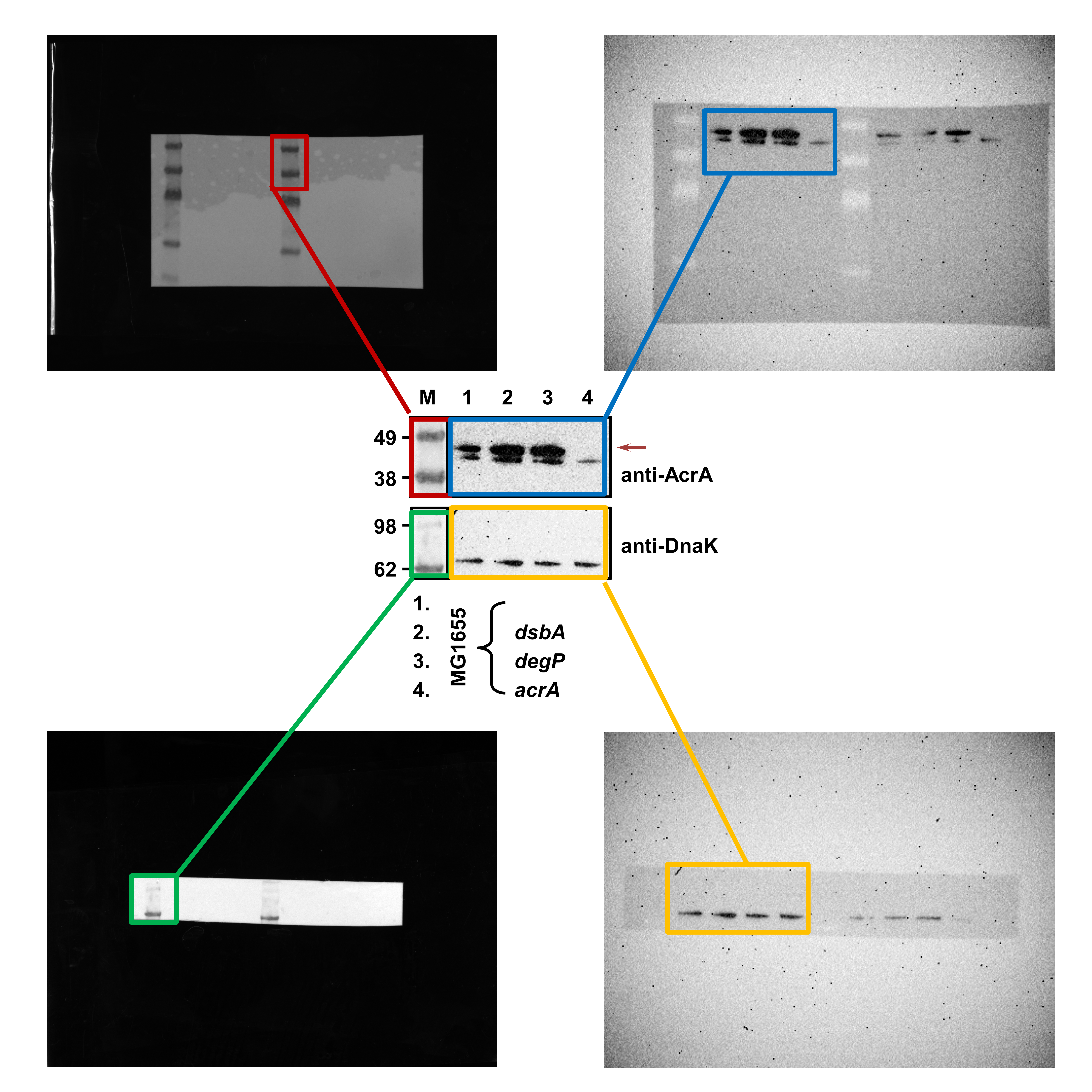

Supplement: Figure 4—source data 4. — The figure included in the paper is shown in the center and relevant bands used for each part of the figure are marked with color-coded boxes on the uncropped immunoblots. [file elife-57974-fig4-data4.zip › Figure 4-source data 4/Figure 4B.tif]

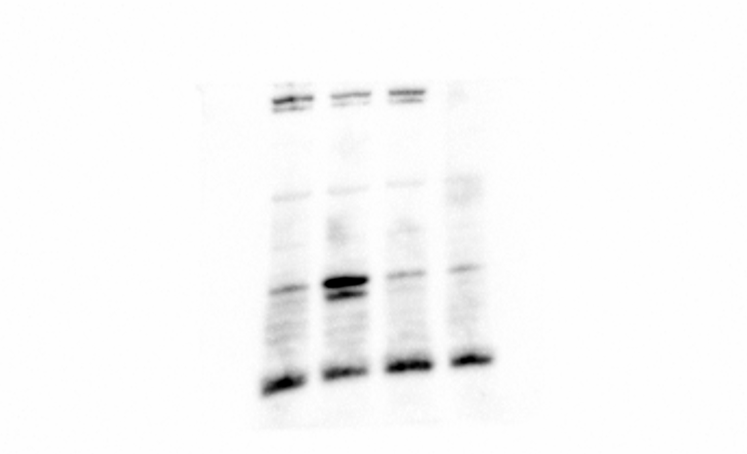

Supplement: Figure 4—source data 5. — ‘Top Panel’ in the file name refers to immunoblots carried out using an anti-TolC antibody, while ‘Bottom Panel’ refers to immunoblots carried out using an anti-DnaK 8E2/2 antibody. ‘Left’ and ‘Right’ in the file names refer to the part of the immunoblot to the left or to the right of the vertical black line shown in the final figure, respectively. [file elife-57974-fig4-data5.zip › Figure 4-source data 5/TopPanel_Right.tif]

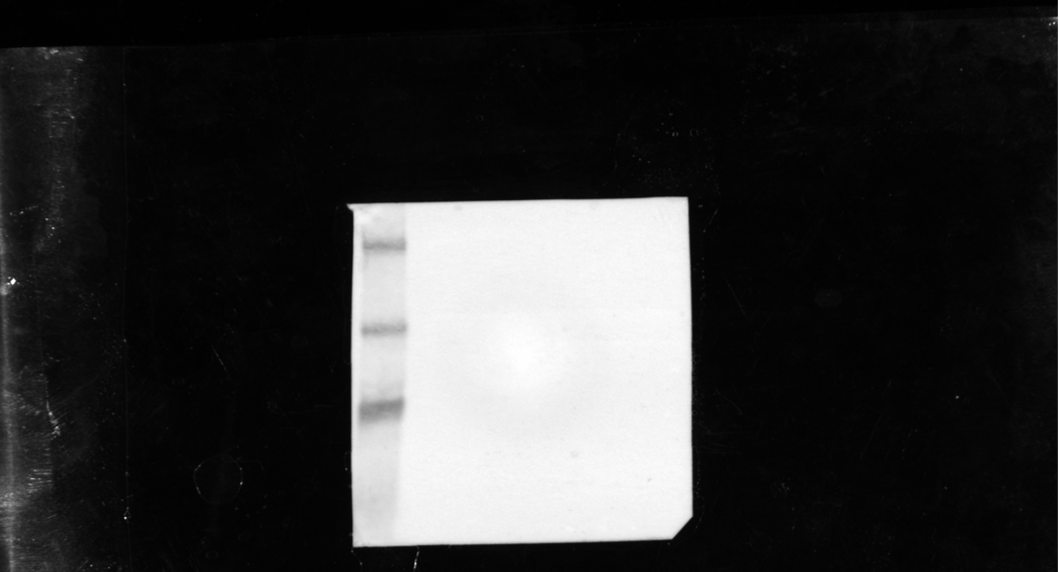

Supplement: Figure 4—source data 5. — ‘Top Panel’ in the file name refers to immunoblots carried out using an anti-TolC antibody, while ‘Bottom Panel’ refers to immunoblots carried out using an anti-DnaK 8E2/2 antibody. ‘Left’ and ‘Right’ in the file names refer to the part of the immunoblot to the left or to the right of the vertical black line shown in the final figure, respectively. [file elife-57974-fig4-data5.zip › Figure 4-source data 5/TopPanel_Left.tif]

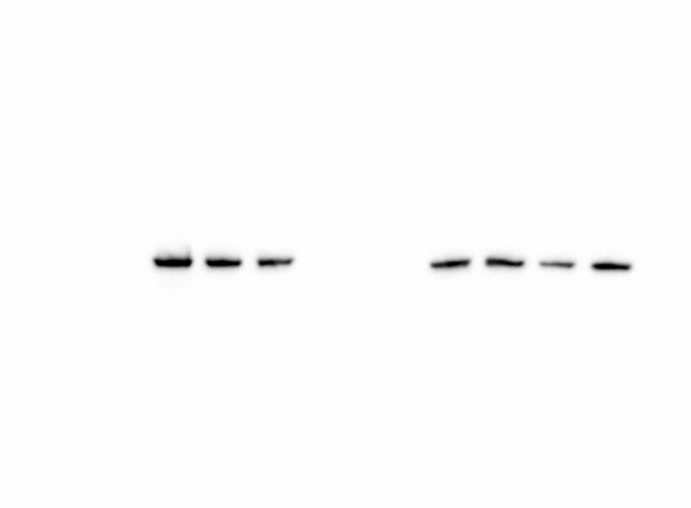

Supplement: Figure 4—source data 5. — ‘Top Panel’ in the file name refers to immunoblots carried out using an anti-TolC antibody, while ‘Bottom Panel’ refers to immunoblots carried out using an anti-DnaK 8E2/2 antibody. ‘Left’ and ‘Right’ in the file names refer to the part of the immunoblot to the left or to the right of the vertical black line shown in the final figure, respectively. [file elife-57974-fig4-data5.zip › Figure 4-source data 5/BottomPanel_Right.tif]

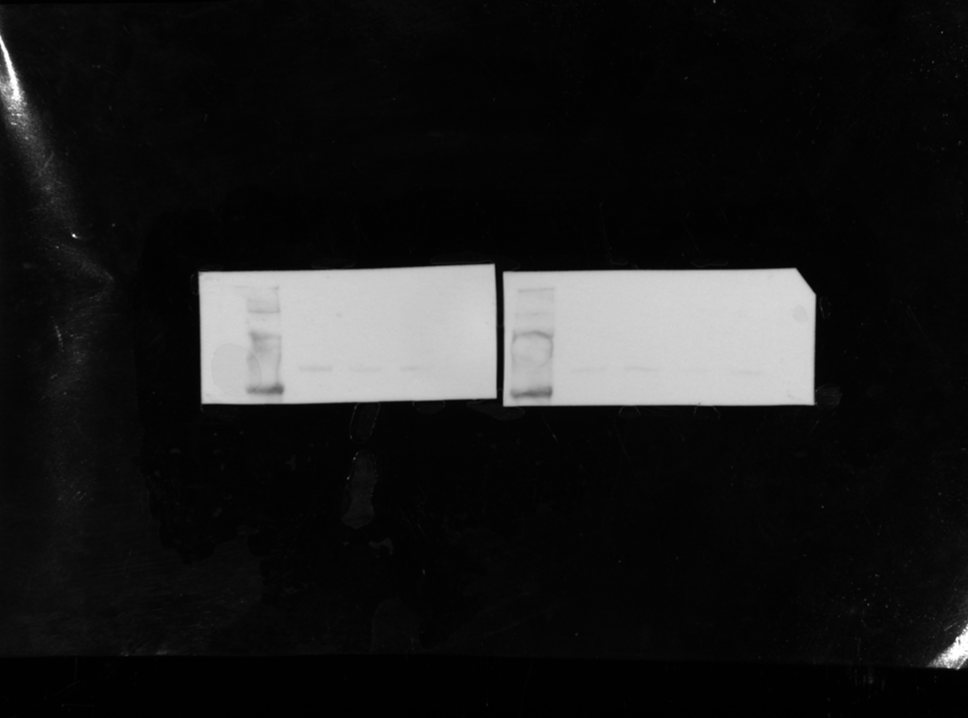

Supplement: Figure 4—source data 5. — ‘Top Panel’ in the file name refers to immunoblots carried out using an anti-TolC antibody, while ‘Bottom Panel’ refers to immunoblots carried out using an anti-DnaK 8E2/2 antibody. ‘Left’ and ‘Right’ in the file names refer to the part of the immunoblot to the left or to the right of the vertical black line shown in the final figure, respectively. [file elife-57974-fig4-data5.zip › Figure 4-source data 5/BottomPanel_Left.tif]

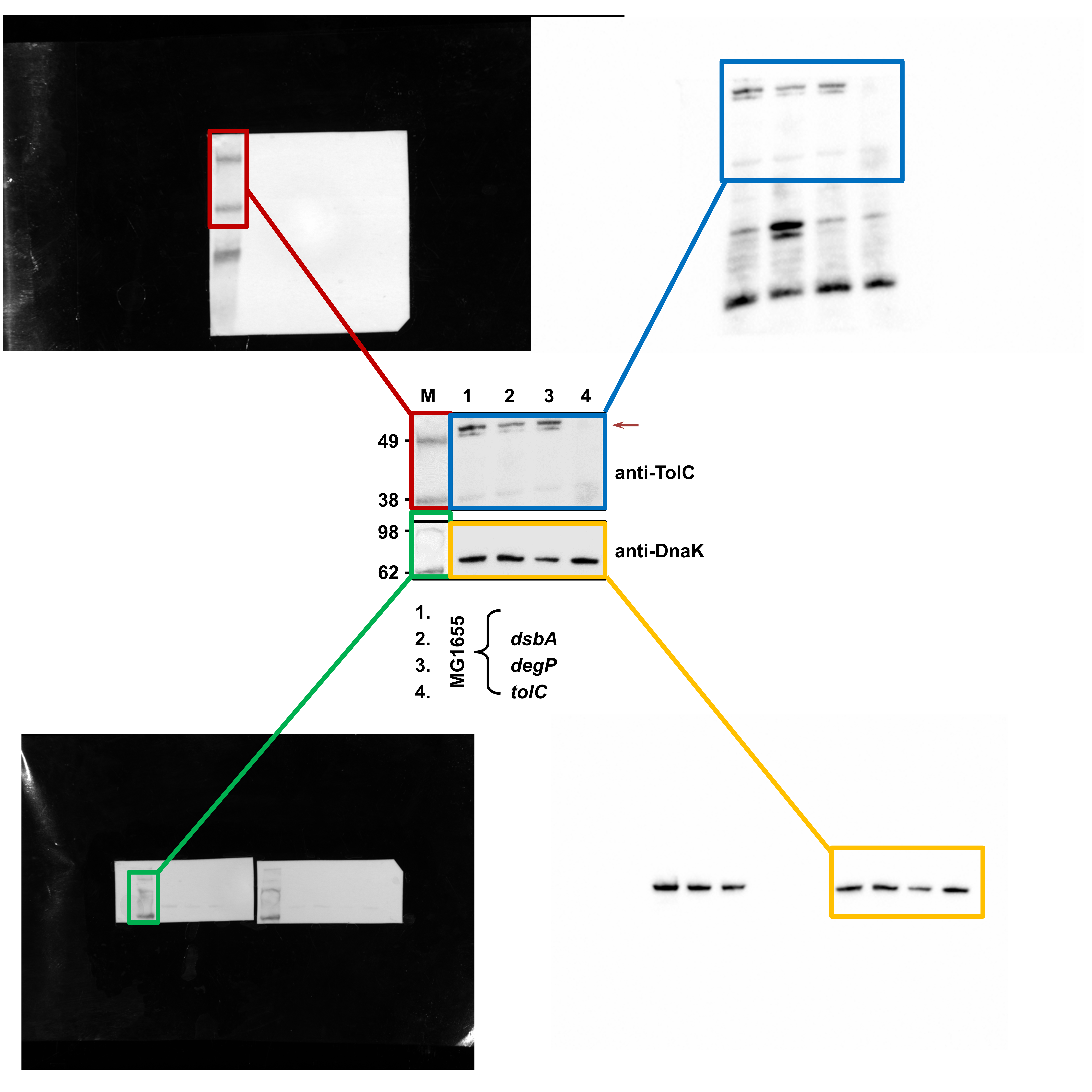

Supplement: Figure 4—source data 6. — The figure included in the paper is shown in the center and relevant bands used for each part of the figure are marked with color-coded boxes on the uncropped immunoblots. [file elife-57974-fig4-data6.zip › Figure 4-source data 6/Figure 4C.tif]

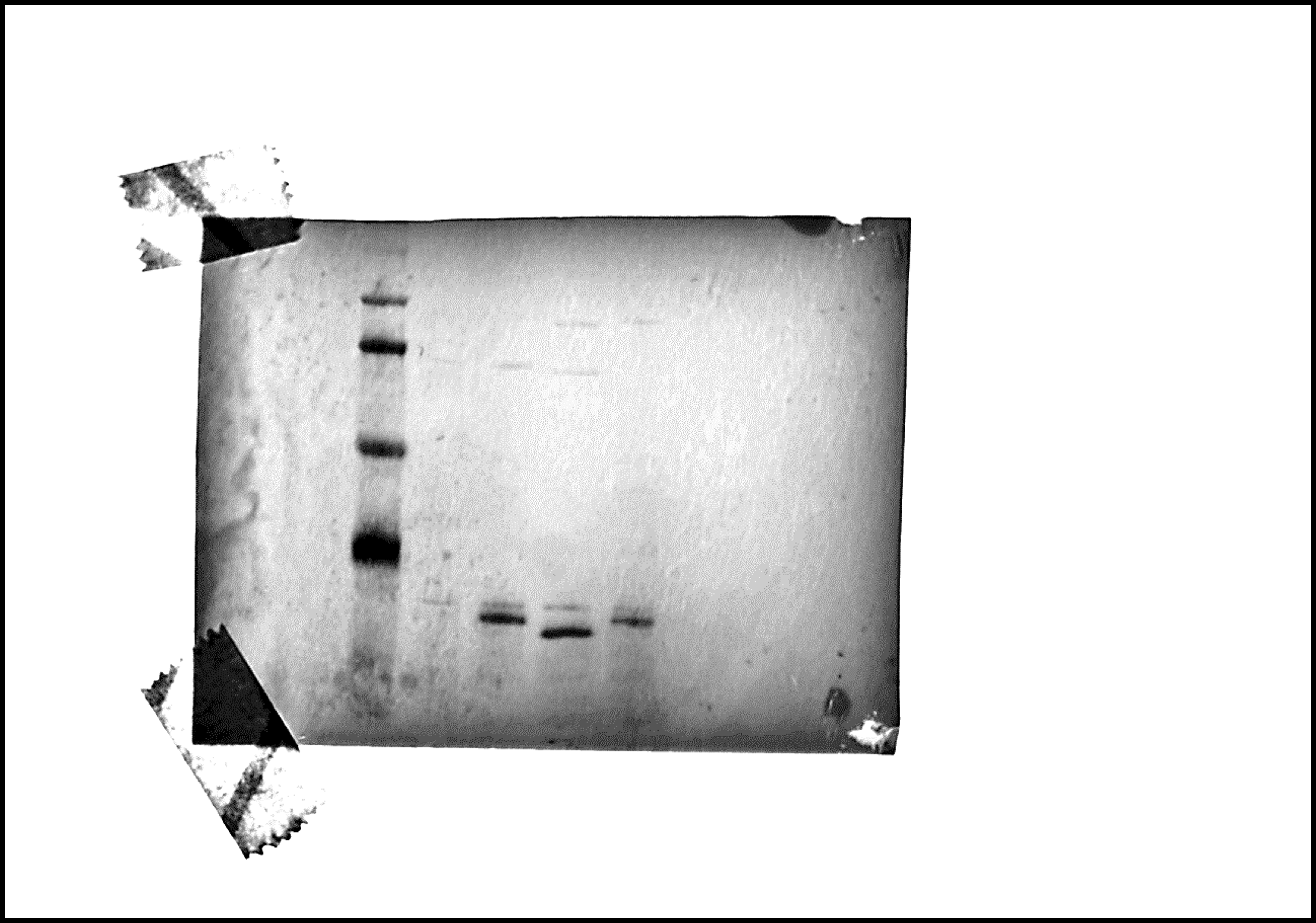

Supplement: Figure 5—source data 1. [file elife-57974-fig5-data1.zip › Figure 5-source data 1/Figure 5C.tif]

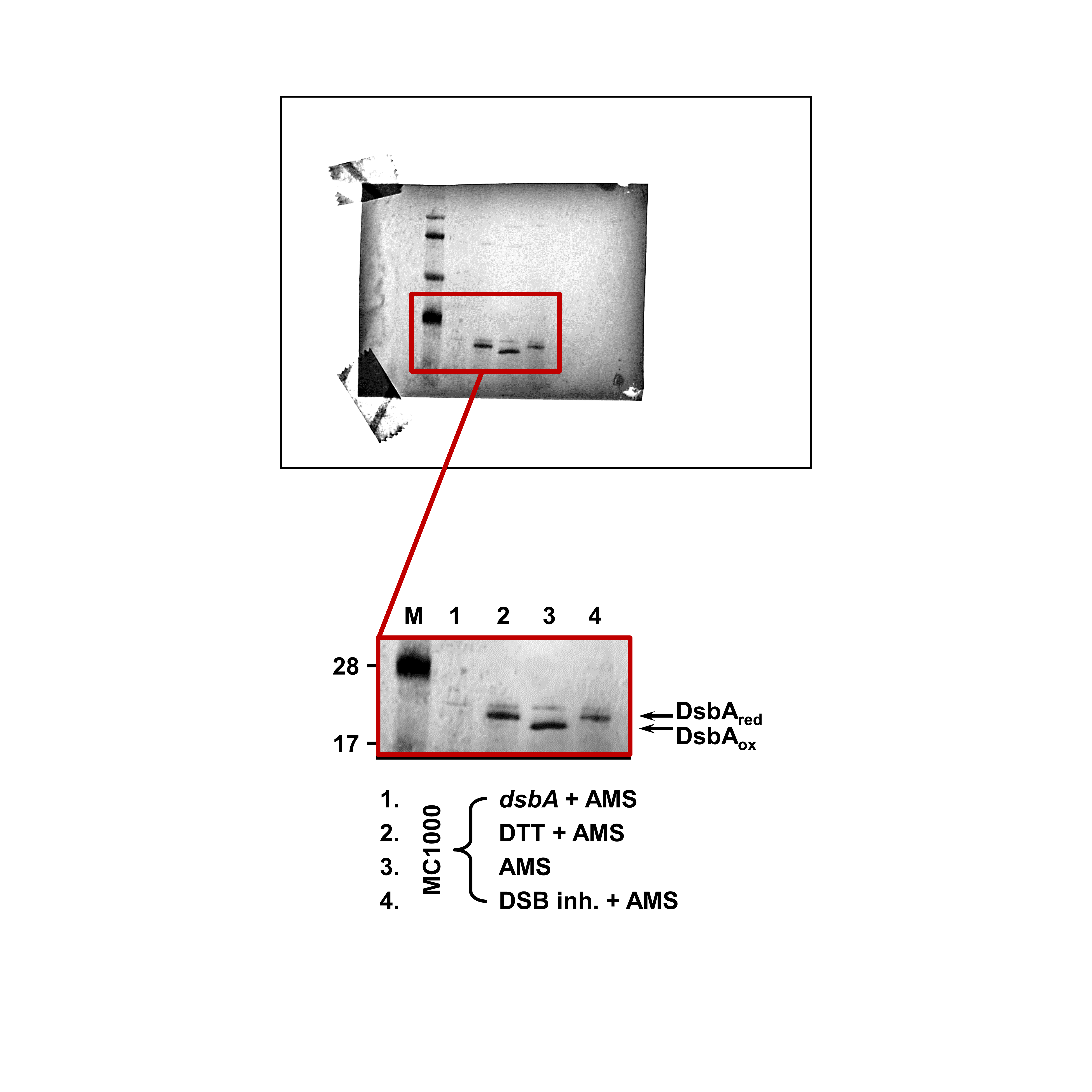

Supplement: Figure 5—source data 2. — The figure included in the paper is shown at the bottom and relevant bands used for each part of the figure are marked with a red box on the uncropped immunoblot. [file elife-57974-fig5-data2.zip › Figure 5-source data 2/Figure 5C.tif]
