# Supplementary material for: Breaking antimicrobial resistance by disrupting extracytoplasmic protein folding
Source: eLife. 2022 Jan 13;11:e57974. doi: 10.7554/eLife.57974 (PMC8863373; doi:10.7554/eLife.57974)
Supplement: Supplementary file 3. [file elife-57974-supp3.docx]

**SUPPLEMENTARY FILE FOR**

**Breaking antimicrobial resistance by disrupting extracytoplasmic protein folding**

R. Christopher D. Furniss^3,†^, Nikol Kadeřábková^1,3,†^, Declan Barker^3^, Patricia Bernal^4^, Evgenia Maslova^5^, Amanda A.A. Antwi^3^, Helen E. McNeil^6^, Hannah L. Pugh^6^, Laurent Dortet^3,7,8,9^, Jessica M.A. Blair^6^, Gerald Larrouy-Maumus^3^, Ronan R. McCarthy^5^, Diego Gonzalez^10^, Despoina A.I. Mavridou^1,2,3,*^

^1^Department of Molecular Biosciences, University of Texas at Austin, Austin, 78712, Texas, USA

^2^John Ring LaMontagne Center for Infectious Diseases, University of Texas at Austin, Austin, 78712, Texas, USA

^3^MRC Centre for Molecular Bacteriology and Infection, Department of Life Sciences, Imperial College London, London, SW7 2AZ, UK

^4^Department of Microbiology, Faculty of Biology, Universidad de Sevilla, Seville, 41012, Spain

^5^Division of Biosciences, Department of Life Sciences, College of Health and Life Sciences, Brunel University London, Uxbridge, UB8 3PH, UK

^6^Institute of Microbiology and Infection, College of Medical and Dental Sciences, University of Birmingham, Birmingham, B15 2TT, UK

^7^Department of Bacteriology-Hygiene, Bicêtre Hospital, Assistance Publique - Hôpitaux de Paris, Le Kremlin-Bicêtre, 94270, France

^8^EA7361 “Structure, Dynamics, Function and Expression of Broad-spectrum β-lactamases", Paris-Sud University, LabEx Lermit, Faculty of Medicine, Le Kremlin-Bicêtre, 94270, France

^9^French National Reference Centre for Antibiotic Resistance, Le Kremlin-Bicêtre, 94270, France

^10^Laboratoire de Microbiologie, Institut de Biologie, Université de Neuchâtel, Neuchâtel, 2000, Switzerland

^*^Correspondence: despoina.mavridou@austin.utexas.edu

^†^These authors have contributed equally to this work

**This file includes:**

Supplementary Tables 1 to 5

Supplementary references

**Supplementary Table 1.** Antibiotic resistance profiles of the clinical isolates tested in this study. The table shows MIC values (µg/mL) for a range of commonly used antibiotics. Values highlighted in pink indicate resistance, as defined by the EUCAST clinical breakpoint guidelines, whilst values highlighted in light blue indicate antibiotics for which there is no EUCAST clinical breakpoint. The remaining values (white cells) indicate sensitivity to the tested antibiotic compound. Cells shaded in grey indicated antibiotic compounds that were not tested for particular clinical isolates. Strains shaded in yellow are multidrug resistant. The following abbreviations are used: AC, amoxicillin; XM, cefuroxime; TZ, ceftazidime; IP, imipenem; AT, aztreonam; PT, piperacillin/tazobactam; GM, gentamicin; CO, colistin; CI, ciprofloxacin; NF, nitrofurantoin; TR, trimethoprim.

| **Strain** | **AC** | **XM** | **TZ** | **IP** | **AT** | **PT** | **GM** | **CO** | **CI** | **NF** | **TR** |
| --- | --- | --- | --- | --- | --- | --- | --- | --- | --- | --- | --- |
| *E. coli* BM16  (*bla*_TEM-1b_ *bla*_KPC-2_) | **>256** | **>256** | **192** | **12** | **>256** | **>64** | 8 | 1 | **>32** | **>512** | **>32** |
| *E. coli* LIL-1  (*bla*_TEM-1_ *bla*_OXA-9_ *bla*_KPC-2_) | **>256** | **>256** | **8** | **3** | **192** | **>64** | 1.5 | 2 | **>32** | 6 | **>32** |
| *E. coli* CNR1790  (*bla*_TEM-15_ *mcr-1*) | **>256** | **>256** | **32** | 0.5 | **16** | <2 | 1 | **4** | **>32** | 16 | **>32** |
| *E. coli* CNR20140385 (*bla*_OXA-48_ *mcr-1*) | **>256** | **96** | 1 | 0.25 | 0.38 | **32** | 2 | **4** | **>32** | 8 | **>32** |
| *E. coli* WI2  (*bla*_OXA-48_ *bla*_KPC-28_ *mcr-1*) | **>256** | **>256** | **>256** | 1.5 | **32** | **>64** | 1.5 | **4** | 0.016 | 6 | 0.25 |
| *E. coli* 1073944  (*mcr-1*) | **>256** | 3 | 0.19 | 0.19 | 0.19 | **64** | **>256** | **16** | - | - | - |
| *E. coli* 41489  (*mcr-1*) | **>256** | **>256** | 1 | 0.19 | 4 | 4 | 1.5 | **16** | - | - | - |
| *E. coli*  (*mcr-1*) | **>256** | 6 | 0.19 | 0.38 | 0.094 | **64** | **16** | **16** | - | - | - |
| *E. coli* 1256822  (*mcr-1.5*) | **>256** | 8 | 0.125 | 0.064 | 0.094 | **32** | **96** | **8** | - | - | - |
| *E. coli* 27841  (*bla*_CTX-M-55_ *mcr-3.2*) | **>256** | **>256** | **16** | 0.19 | **64** | <2 | **32** | **3** | **>32** | 8 | **>32** |
| *E. coli* 1144230  (*bla*_CMY-2_ *mcr-5*) | **>256** | **96** | **12** | 0.5 | **6** | 8 | 1.5 | **4** | 0.025 | 48 | 1 |
| *K. pneumoniae* ST234  (*bla*_SHV-27_ *bla*_KPC-2_) | **>256** | **>256** | **48** | **16** | **128** | **>64** | 0.38 | 2 | 0.047 | **96** | 1 |
| *C. freundii* BM19  (*bla*_KPC-2_) | **>256** | **>256** | **128** | 4 | **64** | **>64** | **24** | 2 | **>32** | 8 | **>32** |
| *E. cloacae* DUB  (*bla*_FRI-1_) | **>256** | **>256** | **16** | **12** | **>256** | **>64** | 1.5 | **>4** | 0.016 | 48 | 0.75 |
| *P. aeruginosa* PA43417  (*bla*_OXA-198_) | **>256** | **>256** | 2 | **>32** | 6 | **32** | **16** | 1 | **>32** | **>256** | **>32** |
| *P. aeruginosa* PAe191  (*bla*_OXA-19_) | **>256** | **>256** | **>256** | 2 | **>256** | **>256** | **>256** | 2 | **>32** | **>256** | **-** |

**Supplementary Table 2.** Bacterial strains used in this study. All listed isolates are clinical strains except for *Escherichia coli* 27841 (ST744), which is an environmental strain. For clinical and environmental isolates, the multi-locus sequence types (ST) are given in parenthesis, where available.

| **Name** | **Description** | **Source** |
| --- | --- | --- |
| ***Escherichia coli*** | | |
| DH5α | F^–^ *end*A1 *gln*V44 *thi*-1 *rec*A1 *rel*A1 *gyr*A96 *deo*R *nup*G *pur*B20 φ80d*lacZ*∆M15 ∆(*lac*ZYA*-arg*F*)*U169 *hsd*R17(r_K_^–^m_K_^+^) λ^–^ | ([1](#_ENREF_1)) |
| CC118λpir | *ara*D Δ(*ara*, *leu*) Δ*lac*Z74 *pho*A20 *gal*K *thi*-1 *rsp*E *rpo*B *arg*E *rec*A1 λ*pir* | ([2](#_ENREF_2)) |
| HB101 | supE44 hsdS20 recA13 ara-14 proA2 lacY1 galK2 rpsL20 xyl-5 mtl-1 | ([3](#_ENREF_3)) |
| MC1000 | *ara*D139 ∆(*ara, leu*)7697 ∆*lac*X74 *gal*U *gal*K *str*A | ([4](#_ENREF_4)) |
| MC1000 *dsbA* | *dsbA::aphA*, Kan^R^ | ([5](#_ENREF_5)) |
| MC1000 *dsbA* *att*Tn*7*::P*tac-dsbA* | *dsbA::aphA* *att*Tn*7*::*dsbA*, Kan^R^ | This study |
| MG1655 | K-12 F^–^ λ*^–^* *ilv*G^–^ *rfb*-50 *rph*-1 | ([6](#_ENREF_6)) |
| MG1655 *dsbA* | *dsbA::aphA*, Kan^R^ | This study |
| MG1655 *dsbA att*Tn*7*::P*tac-dsbA* | *dsbA::aphA* *att*Tn*7*::*dsbA*, Kan^R^ | This study |
| MG1655 *acrA* | *acrA* | This study |
| MG1655 *tolC* | *tolC* | This study |
| MG1655 *degP* | *degP::strAB*, Str^R^ | This study |
| **Clinical / environmental isolates** | | |
| *Escherichia coli* BM16 | *bla*_TEM-1b_ *bla*_KPC-_*_2_* | ([7](#_ENREF_7)) |
| *Escherichia coli* LIL-1 | *bla*_TEM-1_ *bla*_OXA-9_ *bla*_KPC-2_ | ([7](#_ENREF_7)) |
| *Escherichia coli* CNR1790 | *bla*_TEM-15_ *mcr-1* | ([8](#_ENREF_8)) |
| *Escherichia coli* CNR20140385 | *bla*_OXA-48_ *mcr-1* | ([8](#_ENREF_8)) |
| *Escherichia coli* WI2 (ST1288) | *bla*_OXA-48_ *bla*_KPC-28_ *mcr-1* | ([9](#_ENREF_9)) |
| *Escherichia coli* 1073944 (ST117) | *mcr-1* | ([10](#_ENREF_10)) |
| *Escherichia coli* 41489 | *mcr-1* | ([8](#_ENREF_8)) |
| *Escherichia coli* | *mcr-1* | ([8](#_ENREF_8)) |
| *Escherichia coli* 1256822 (ST48) | *mcr-1.5* | ([10](#_ENREF_10)) |
| *Escherichia coli* 27841 (ST744) | *bla*_CTX-M-55_ *mcr-3.2* | ([11](#_ENREF_11)) |
| *Escherichia coli* 1144230 (ST641) | *bla*_CMY-2_ *mcr-5* | ([10](#_ENREF_10)) |
| *Klebsiella pneumoniae* (ST234) | *bla*_SHV-27_ *bla*_KPC-2_ | ([12](#_ENREF_12)) |
| *Citrobacter freundii* BM19 | *bla*_KPC-2_ | ([7](#_ENREF_7)) |
| *Enterobacter cloacae* DUB | *bla*_FRI-1_ | ([13](#_ENREF_13)) |
| *Pseudomonas aeruginosa* PA43417 | *bla*_OXA-198_ | ([14](#_ENREF_14)) |
| *Pseudomonas aeruginosa* PA43417 *dsbA1* | *dsbA1 bla*_OXA-198_ | This study |
| *Pseudomonas aeruginosa* PAe191 | *bla*_OXA-19_ | ([15](#_ENREF_15)) |
| *Pseudomonas aeruginosa* PAe191 *dsbA1* | *dsbA1 bla*_OXA-19_ | This study |

**Supplementary Table 3.** Plasmids used in this study.

| **Name** | **Description** | **Source** |
| --- | --- | --- |
| pDM1 | pDM1 vector (GenBank MN128719), p15A *ori*, P*tac* promoter, MCS, Tet^R^ | Lab stock |
| pDM1-*bla*_L2-1_ | *bla*_L2-1_ cloned into pDM1, Tet^R^ | This study |
| pDM1-*bla*_GES-1_ | *bla*_GES-1_ cloned into pDM1, Tet^R^ | This study |
| pDM1-*bla*_GES-2_ | *bla*_GES-2_ cloned into pDM1, Tet^R^ | This study |
| pDM1-*bla*_GES-11_ | *bla*_GES-11_ cloned into pDM1, Tet^R^ | This study |
| pDM1-*bla*_SHV-27_ | *bla*_SHV-27_ cloned into pDM1, Tet^R^ | This study |
| pDM1-*bla*_OXA-4_ | *bla*_OXA-4_ cloned into pDM1, Tet^R^ | This study |
| pDM1-*bla*_OXA-10_ | *bla*_OXA-10_ cloned into pDM1, Tet^R^ | This study |
| pDM1-*bla*_OXA-198_ | *bla*_OXA-198_ cloned into pDM1, Tet^R^ | This study |
| pDM1-*bla*_FRI-1_ | *bla*_FRI-1_ cloned into pDM1, Tet^R^ | This study |
| pDM1-*bla*_L1-1_ | *bla*_L1-1_ cloned into pDM1, Tet^R^ | This study |
| pDM1-*bla*_KPC-2_ | *bla*_KPC-2_ cloned into pDM1, Tet^R^ | This study |
| pDM1-*bla*_KPC-3_ | *bla*_KPC-3_ cloned into pDM1, Tet^R^ | This study |
| pDM1-*bla*_SME-1_ | *bla*_SME-1_ cloned into pDM1, Tet^R^ | This study |
| pDM1-*mcr-1* | *mcr-1* cloned into pDM1, Tet^R^ | This study |
| pDM1-*mcr-3* | *mcr-3* cloned into pDM1, Tet^R^ | This study |
| pDM1-*mcr-4* | *mcr-4* cloned into pDM1, Tet^R^ | This study |
| pDM1-*mcr-5* | *mcr-5* cloned into pDM1, Tet^R^ | This study |
| pDM1-*mcr-8* | *mcr-8* cloned into pDM1, Tet^R^ | This study |
| pDM1-*bla*_L2-1_-StrepII | *bla*_L2-1_ encoding L2-1 with a C-terminal StrepII tag cloned into pDM1, Tet^R^ | This study |
| pDM1-*bla*_GES-1_-StrepII | *bla*_GES-1_ encoding GES-1 with a C-terminal StrepII tag cloned into pDM1, Tet^R^ | This study |
| pDM1-StrepII-*bla*_OXA-4_ | *bla*_OXA-4_ encoding OXA-4 with an N-terminal StrepII tag cloned into pDM1, Tet^R^ | This study |
| pDM1-*bla*_OXA-10_-StrepII | *bla*_OXA-10_ encoding OXA-10 with a C-terminal StrepII tag cloned into pDM1, Tet^R^ | This study |
| pDM1-*bla*_OXA-198_-StrepII | *bla*_OXA-198_ encoding OXA-198 with a C-terminal StrepII tag cloned into pDM1, Tet^R^ | This study |
| pDM1-*bla*_FRI-1_-StrepII | *bla*_FRI-1_ encoding FRI-1 with a C-terminal StrepII tag cloned into pDM1, Tet^R^ | This study |
| pDM1-*bla*_L1-1_-StrepII | *bla*_L1-1_ encoding L1-1 with a C-terminal StrepII tag cloned into pDM1, Tet^R^ | This study |
| pDM1-*bla*_KPC-3_-StrepII | *bla*_KPC-3_ encoding KPC-3 with a C-terminal StrepII tag cloned into pDM1, Tet^R^ | This study |
| pDM1-*mcr-1*-StrepII | *bla*_MCR-1_ encoding MCR-1 with a C-terminal StrepII tag cloned into pDM1, Tet^R^ | This study |
| pDM1-*mcr-3*-StrepII | *bla*_MCR-3_ encoding MCR-3 with a C-terminal StrepII tag cloned into pDM1, Tet^R^ | This study |
| pDM1-*mcr-4*-StrepII | *bla*_MCR-4_ encoding MCR-4 with a C-terminal StrepII tag cloned into pDM1, Tet^R^ | This study |
| pDM1-*mcr-5*-StrepII | *bla*_MCR-5_ encoding MCR-5 with a C-terminal StrepII tag cloned into pDM1, Tet^R^ | This study |
| pDM1-*mcr-8*-StrepII | *bla*_MCR-8_ encoding MCR-8 with a C-terminal StrepII tag cloned into pDM1, Tet^R^ | This study |
| pGRG25 | Encodes a Tn*7* transposon and *tnsABCD* under the control of P*araB*, thermosensitive pSC101 *ori*, Amp^R^ | ([16](#_ENREF_16)) |
| pGRG25-P*tac::dsbA* | P*tac::dsbA* fragment cloned within the Tn*7* of pGRG25; when inserted into the chromosome and the plasmid cured, the strain expresses DsbA upon IPTG induction, Amp^R^ | This study |
| pSLTS | Thermosensitive pSC101*ori,* P*araB* for λ-Red, P*tetR* for I-SceI, Amp^R^ | ([17](#_ENREF_17)) |
| pUltraGFP-GM | Constitutive sfGFP expression from a strong Biofab promoter, p15A *ori*, Gent^R^ | ([18](#_ENREF_18)) |
| pKD4 | Conditional oriRγ *ori*, (template for the *aphA* cassette), Amp^R^ | ([19](#_ENREF_19)) |
| pCB112 | Inducible *lacZ* expression under the control of the P_lac_ promoter, pBR322 *ori*, Cam^R^ | ([20](#_ENREF_20)) |
| pKNG101 | Gene replacement suicide vector, *ori*R6K, *oriT*RK2, *sacB*, (template for the *strAB* cassette), Str^R^ | ([21](#_ENREF_21)) |
| pKNG101-*dsbA1* | PCR fragment containing the regions upstream and downstream *P. aeruginosa dsbA1* cloned in pKNG101; when inserted into the chromosome the strain is a merodiploid for *dsbA1* mutant, Str^R^ | This study |
| pRK600 | Helper plasmid, ColE1 ori, mobRK2, traRK2, Cam^R^ | ([22](#_ENREF_22)) |
| pMA-T *mcr-3* | GeneArt® cloning vector containing *mcr-3*, ColE1 *ori*, (template for *mcr-3*), Amp^R^ | This study |
| pMK-T *mcr-8* | GeneArt® cloning vector containing *mcr-8*, ColE1 *ori*, (template for *mcr-8*), Kan^R^ | This study |

**Supplementary Table 4.** Oligonucleotide primers used in this study. The “Brief description” column provides basic information on the primer design (restriction enzyme used for cloning, encoded protein or gene replaced by antibiotic resistance cassette, forward or reverse orientation of the primer (F or R); QC stands for QuickChange primers and SQ stands for sequencing primers).

| **Number** | **Brief description** | **Sequence (5ˊ-3ˊ)** |
| --- | --- | --- |
| P1 | SacI.L2.F | ctggagctcctcgcccgtcgccgatt |
| P2 | XmaI.L2.R | ctgcccgggtcatccgatcaaccggtcggca |
| P3 | SacI.GES.F | ctggagctccgcttcattcacgcac |
| P4 | XmaI.GES.R | ctgcccgggctatttgtccgtgctcaggatg |
| P5 | SacI.SHV.F | ctggagctccgttatattcgcctgtg |
| P6 | XmaI.SHV.R | ctgcccgggttagcgttgccagtgctcga |
| P7 | SacI.OXA-4.F | ctggagctcaaaaacacaatacatataacttcgc |
| P8 | KpnI.OXA-4.R | cagggtaccttataaatttagtgtgtttagaatggtg |
| P9 | SacI.OXA-10.F | ctggagctcaaaacatttgccgcatatgtaattatcgc |
| P10 | KpnI.OXA-10.R | cagggtaccttagccaccaatgatgccctc |
| P11 | NdeI.OXA-198.F | actgcatatgcataaacacatgagtaagctcttc |
| P12 | KpnI.OXA-198.R | ctgggtaccttattcgatgatcccctttgctt |
| P13 | SacI.FRI-1.F | ctggagctctttttttttaaaaaaggtgcaagtac |
| P14 | XmaI.FRI-1.R | ctgcccgggttatttataacttccataaactgcctttatagc |
| P15 | SacI.L1.F | ctggagctccgttctaccctgctcgc |
| P16 | XhoI.L1.R | actgagctctcagcgggccccggccgt |
| P17 | SacI.KPC.F | ctggagctctcactgtatcgccgtc |
| P18 | KpnI.KPC.R | ctgccatggttactgcccgttgacgccca |
| P19 | SacI.SME-1.F | ctggagctctcaaacaaagtaaattttaaaacgg |
| P20 | XmaI.SME-1.R | ctgcccgggttaatcaattgcctgaattgcaatacg |
| P21 | SacI.MCR-1.F | ctggagctcatgcagcatacttctgtgtggtac |
| P22 | XmaI.MCR-1.R | ctgcccgggtcagcggatgaatgcggtgc |
| P23 | NdeI.MCR-3.F | ctgatacatatgccttcccttataaaaataaaaattgttccg |
| P24 | XmaI.MCR-3.R | cagcccgggttattgaacattacgacattgactgaaaatatctag |
| P25 | SacI.MCR-4.F | ctggagctccgtgctgacgagatttaaaaccc |
| P26 | XmaI.MCR-4.R | ctgcccgggttaaccgcggcagcgggcaaaaatatc |
| P27 | SacI.MCR-5.F | ctggagctccggttgtctgcatttatcac |
| P28 | XmaI.MCR-5.R | ctgcccgggtcattgtggttgtccttttctg |
| P29 | SacI.MCR-8.F | ctggagctcttcaagtatcttttatctttcaaact aacc |
| P30 | XmaI.MCR-8.R | ctgcccgggctaaccattcccatctgttttctc |
| P31 | QC.GES5-GES1.F | aaagagccggagatgggcgacaacacacctg |
| P32 | QC.GES5-GES1.R | caggtgtgttgtcgcccatctccggctcttt |
| P33 | QC.KPC2-KPC3.F | ctaacaaggatgacaagtacagcgaggccgtcatc |
| P34 | QC.KPC2-KPC3.R | gatgacggcctcgctgtacttgtcatccttgttag |
| P35 | XmaI.StrepII.L2.R | ctgcccgggttatttttcaaattgcggatggctccaagcgctccctccgatcaaccggtcggca |
| P36 | XmaI.StrepII.GES.R | ctgcccgggctatttttcaaattgcggatggctccaagcgctccctttgtccgtgctcaggatgag |
| P37 | OXA-4.body.F | tcaacagatatctctactgttgca |
| P38 | OXA-4.StrepII.R | tgcaacagtagagatatctgttgatttttcaaattgcggatggctccaagcgctccctgcactggcgctgctgta |
| P39 | KpnI.StrepII.OXA-10.R | cagggtaccttatttttcaaattgcggatggctccaagcgctcccgccaccaatgatgccctcacttg |
| P40 | KpnI.StrepII.OXA-198.R | ctgggtaccttatttttcaaattgcggatggctccaagcgctcccttcgatgatcccctttgcttg |
| P41 | XmaI.StrepII.FRI-1.R | ctgcccgggttatttttcaaattgcggatggctccaagcgctccctttataacttccataaactgcctttatagc |
| P42 | KpnI.StrepII.L1.R | gggggtacctcatttttcaaattgcggatggctccaagcgctcccgcgggccccggccgtttccttggccaactgc |
| P43 | KpnI.StrepII.KPC.R | ctgccatggttatttttcaaattgcggatggctccaagcgctcccctgcccgttgacgcccaatc |
| P44 | XmaI.StrepII.MCR-1.R | cagcccgggttatttttcaaattgcggatggctccaagcgctcccgcggatgaatgcggtgcggt |
| P45 | XmaI.StrepII.MCR-3.R | cagcccgggttatttttcaaattgcggatggctccaagcgctcccttgaacattacgacattgactgaaaatatctag |
| P46 | XmaI.StrepII.MCR-4.R | ctgcccgggctatttttcaaattgcggatggctccaagcgctcccaccgcggcagcgggcaaaaatatc |
| P47 | XmaI.StrepII.MCR-5.R | ctgcccgggctatttttcaaattgcggatggctccaagcgctcccttgtggttgtccttttctgca |
| P48 | XmaI.StrepII.MCR-8.R | ctgcccgggctatttttcaaattgcggatggctccaagcgctcccaccattcccatctgttttctctcttac |
| P49 | NotI.Ptac.EcDsbA.F | ctggcggccgctgacaattaatcatcggctcgtataatgtgtggaattgtgactagtcgaggtccaggacctcggatcgctaagataggatgattgtatgaaaaagatttggctggc |
| P50 | XhoI.EcDsbA.R | ctgctcgagttattttttctcggacagatatttc |
| P51 | EcdsbA::aphA.F | atgaaaaagatttggctggcgctggctggtttagttttagcgtttagcgcgtgtaggctggagctgcttc |
| P52 | EcdsbA::aphA.R | ttattttttctcggacagatatttcactgtatcagcatactgctgaacaagggaattagccatggtccat |
| P53 | EcacrA::aphA.F | atgaacaaaaacagagggtttacgcctctggcggtcgttctggtgtaggctggagctgcttc |
| P54 | EcacrA::aphA.R | ttaagacttggactgttcaggctgagcaccgcttgcggcttggggaattagccatggtccat |
| P55 | EctolC::aphA.F | ttttacagtttgatcgcgctaaatactgcttcaccacaaggaatgcaagtgtaggctggagctgcttc |
| P56 | EctolC::aphA.R | tcgtcgtcatcagttacggaaagggttatgatgggaattagccatggtcc |
| P57 | EcdegP::strAB.F | atgaaaaaaaccacattagcactgagtgcactggctctgagtttaggtttggaactgcacattcgggatatttctc |
| P58 | EcdegP::strAB.R | ttactgcattaacaggtagatggtgctgtcgccgcgctgaatgttgagtgccaggccggatctagatatctagtatga |
| P59 | SQ.dsbA1.Paeruginosa.F | tacctgctcaagcagatgcatg |
| P60 | SQ.dsbA1.Paeruginosa.R | ggtgttcatgtcgcccatca |
| P61 | XbaI.dsbA1.F | ggttcctctagagcctacttcgccagccagaa |
| P62 | dsbA1.body.R | ctacttcttgttacgcatcgttcactc |
| P63 | dsbA1.body.F | atgcgtaacaagaagtaggcaaggtga |
| P64 | BamHI.dsbA1.R | aattaaggatcctcatcactaccaccagcgcg |

**Supplementary Table 5.** Sources of genomic DNA used for amplification of β-lactamase and MCR genes in this study.

| **Strain** | **Gene(s)** | **Source** |
| --- | --- | --- |
| *Stenotrophomonas maltophilia* ATCC 13637 | *bla*_L2-1_ *bla*_L1-1_ | ATCC |
| *Pseudomonas aeruginosa* GW-1 | *bla*_GES-2_ | ([23](#_ENREF_23)) |
| *Enterobacter cloacae* CHE-2 | *bla*_GES-5_ | ([24](#_ENREF_24)) |
| *Acinetobacter baumannii* K45 | *bla*_GES-11_ | ([25](#_ENREF_25)) |
| *Klebsiella pneumoniae* ST234 | *bla*_SHV-27_ *bla*_KPC-2_ | ([12](#_ENREF_12)) |
| *Pseudomonas aeruginosa* SOF1 | *bla*_OXA-4_ | ([26](#_ENREF_26)) |
| *Pseudomonas aeruginosa* PU21 | *bla*_OXA-10_ | ([27](#_ENREF_27)) |
| *Pseudomonas aeruginosa* PA41437 | *bla*_OXA-198_ | ([14](#_ENREF_14)) |
| *Enterobacter cloacae* DUB | *bla*_FRI-1_ | ([13](#_ENREF_13)) |
| *Serratia marcescens* | *bla*_SME-1_ | ([12](#_ENREF_12)) |
| *Escherichia coli* CNR1790 | *mcr-1* | ([8](#_ENREF_8)) |
| *Shewanella bicestrii* JAB-1 | *mcr-4* | ([28](#_ENREF_28)) |
| *Escherichia coli* 1144230 | *mcr-5* | ([10](#_ENREF_10)) |

**SUPPLEMENTARY REFERENCES**

1. Hanahan D. In: Glover DM, editor. DNA Cloning: A Practical Approach. 1: IRL Press, McLean, Virginia; 1985. p. 109.

2. Herrero M, de Lorenzo V, Timmis KN. Transposon vectors containing non-antibiotic resistance selection markers for cloning and stable chromosomal insertion of foreign genes in Gram-negative bacteria. J. Bacteriol. 1990;172(11):6557-67.

3. Boyer HW, Roulland-Dussoix D. A complementation analysis of the restriction and modification of DNA in *Escherichia coli*. J. Mol. Biol. 1969;41(3):459-72.

4. Casadaban MJ, Cohen SN. Analysis of gene control signals by DNA fusion and cloning in *Escherichia coli*. J. Mol. Biol. 1980;138(2):179-207.

5. Kadokura H, Tian H, Zander T, Bardwell JC, Beckwith J. Snapshots of DsbA in action: detection of proteins in the process of oxidative folding. Science. 2004;303(5657):534-7.

6. Blattner FR, Plunkett G, 3rd, Bloch CA, Perna NT, Burland V, Riley M, et al. The complete genome sequence of *Escherichia coli* K-12. Science. 1997;277(5331):1453-62.

7. Dortet L, Brechard L, Poirel L, Nordmann P. Rapid detection of carbapenemase-producing *Enterobacteriaceae* from blood cultures. Clin. Microbiol. Infect. 2014;20(4):340-4.

8. Dortet L, Bonnin RA, Pennisi I, Gauthier L, Jousset AB, Dabos L, et al. Rapid detection and discrimination of chromosome- and MCR-plasmid-mediated resistance to polymyxins by MALDI-TOF MS in *Escherichia coli*: the MALDIxin test. J. Antimicrob. Chemother. 2018;73(12):3359-67.

9. Beyrouthy R, Robin F, Lessene A, Lacombat I, Dortet L, Naas T, et al. MCR-1 and OXA-48 *in vivo* acquisition in KPC-producing *Escherichia coli* after colistin treatment. Antimicrob. Agents Chemother. 2017;61(8).

10. Wise MG, Estabrook MA, Sahm DF, Stone GG, Kazmierczak KM. Prevalence of *mcr*-type genes among colistin-resistant *Enterobacteriaceae* collected in 2014-2016 as part of the INFORM global surveillance program. PloS One. 2018;13(4):e0195281.

11. Haenni M, Beyrouthy R, Lupo A, Chatre P, Madec JY, Bonnet R. Epidemic spread of *Escherichia coli* ST744 isolates carrying *mcr-3* and *bla*_CTX-M-55_ in cattle in France. J. Antimicrob. Chemother. 2018;73(2):533-6.

12. Nordmann P, Poirel L, Dortet L. Rapid detection of carbapenemase-producing *Enterobacteriaceae*. Emerg. Infect. Dis. 2012;18(9):1503-7.

13. Dortet L, Poirel L, Abbas S, Oueslati S, Nordmann P. Genetic and biochemical characterization of FRI-1, a carbapenem-hydrolyzing class A β-lactamase from *Enterobacter cloacae*. Antimicrob. Agents Chemother. 2015;59(12):7420-5.

14. El Garch F, Bogaerts P, Bebrone C, Galleni M, Glupczynski Y. OXA-198, an acquired carbapenem-hydrolyzing class D β-lactamase from *Pseudomonas aeruginosa*. Antimicrob. Agents Chemother. 2011;55(10):4828-33.

15. Mugnier P, Casin I, Bouthors AT, Collatz E. Novel OXA-10-derived extended-spectrum β-lactamases selected *in vivo* or *in vitro*. Antimicrob. Agents Chemother. 1998;42(12):3113-6.

16. McKenzie GJ, Craig NL. Fast, easy and efficient: site-specific insertion of transgenes into enterobacterial chromosomes using Tn*7* without need for selection of the insertion event. BMC Microbiol. 2006;6:39.

17. Kim J, Webb AM, Kershner JP, Blaskowski S, Copley SD. A versatile and highly efficient method for scarless genome editing in *Escherichia coli* and *Salmonella enterica*. BMC Biotechnol. 2014;14:84.

18. Mavridou DA, Gonzalez D, Clements A, Foster KR. The pUltra plasmid series: a robust and flexible tool for fluorescent labeling of *Enterobacteria*. Plasmid. 2016;87-88:65-71.

19. Datsenko KA, Wanner BL. One-step inactivation of chromosomal genes in *Escherichia coli* K-12 using PCR products. Proc. Natl. Acad. Sci. U. S. A. 2000;97(12):6640-5.

20. Paradis-Bleau C, Kritikos G, Orlova K, Typas A, Bernhardt TG. A genome-wide screen for bacterial envelope biogenesis mutants identifies a novel factor involved in cell wall precursor metabolism. PLoS Genet. 2014;10(1):e1004056.

21. Kaniga K, Delor I, Cornelis GR. A wide-host-range suicide vector for improving reverse genetics in Gram-negative bacteria: inactivation of the *blaA* gene of *Yersinia enterocolitica*. Gene. 1991;109(1):137-41.

22. Kessler B, Delorenzo V, Timmis KN. A general system to integrate *lacZ* fusions into the chromosomes of Gram-negative eubacteria: regulation of the *Pm* Promoter of the *TOL* plasmid studied with all controlling elements in monocopy. Mol. Gen. Genet. 1992;233(1-2):293-301.

23. Poirel L, Weldhagen GF, Naas T, De Champs C, Dove MG, Nordmann P. GES-2, a class A β-lactamase from *Pseudomonas aeruginosa* with increased hydrolysis of imipenem. Antimicrob. Agents Chemother. 2001;45(9):2598-603.

24. Poirel L, Carrer A, Pitout JD, Nordmann P. Integron mobilization unit as a source of mobility of antibiotic resistance genes. Antimicrob. Agents Chemother. 2009;53(6):2492-8.

25. Bonnin RA, Rotimi VO, Al Hubail M, Gasiorowski E, Al Sweih N, Nordmann P, et al. Wide dissemination of GES-type carbapenemases in *Acinetobacter baumannii* isolates in Kuwait. Antimicrob. Agents Chemother. 2013;57(1):183-8.

26. Aubert D, Poirel L, Chevalier J, Leotard S, Pages JM, Nordmann P. Oxacillinase-mediated resistance to cefepime and susceptibility to ceftazidime in *Pseudomonas aeruginosa*. Antimicrob. Agents Chemother. 2001;45(6):1615-20.

27. Dortet L, Poirel L, Nordmann P. Rapid detection of carbapenemase-producing *Pseudomonas spp*. J.Clin. Microbiol. 2012;50(11):3773-6.

28. Jousset AB, Dabos L, Bonnin RA, Girlich D, Potron A, Cabanel N, et al. CTX-M-15-producing *Shewanella* species clinical isolate expressing OXA-535, a chromosome-encoded OXA-48 variant, putative progenitor of the plasmid-encoded OXA-436. Antimicrob. Agents Chemother. 2018;62(1).
