# Supplementary material for: Breaking antimicrobial resistance by disrupting extracytoplasmic protein folding
Source: eLife. 2022 Jan 13;11:e57974. doi: 10.7554/eLife.57974 (PMC8863373; doi:10.7554/eLife.57974)
Supplement: Transparent reporting form [file elife-57974-transrepform1.docx]

***eLife’s* transparent reporting form**

We encourage authors to provide detailed information *within their submission* to facilitate the interpretation and replication of experiments. Authors can upload supporting documentation to indicate the use of appropriate reporting guidelines for health-related research (see [EQUATOR Network](http://www.equator-network.org/%20)), life science research (see the [BioSharing Information Resource](https://biosharing.org/)), or the [ARRIVE guidelines](http://www.plosbiology.org/article/info:doi/10.1371/journal.pbio.1000412) for reporting work involving animal research. Where applicable, authors should refer to any relevant reporting standards documents in this form.

If you have any questions, please consult our Journal Policies and/or contact us: [editorial@elifesciences.org](mailto:editorial@elifesciences.org).

**Sample-size estimation**

- You should state whether an appropriate sample size was computed when the study was being designed
- You should state the statistical method of sample size computation and any required assumptions
- If no explicit power analysis was used, you should describe how you decided what sample (replicate) size (number) to use

Please outline where this information can be found within the submission (e.g., sections or figure legends), or explain why this information doesn’t apply to your submission:

No sampling was performed for the purpose of this manuscript.

**Replicates**

- You should report how often each experiment was performed
- You should include a definition of biological versus technical replication
- The data obtained should be provided and sufficient information should be provided to indicate the number of independent biological and/or technical replicates
- If you encountered any outliers, you should describe how these were handled
- Criteria for exclusion/inclusion of data should be clearly stated
- High-throughput sequence data should be uploaded before submission, with a private link for reviewers provided (these are available from both GEO and ArrayExpress)

Please outline where this information can be found within the submission (e.g., sections or figure legends), or explain why this information doesn’t apply to your submission:

Information on biological and technical replication can be found in each figure legend. A definition of biological versus technical replication as well as information on how outliers were handled can be found in the Materials and Methods section of the manuscript under the heading *Statistical analysis of experimental data*. No high-throughput sequence data were acquired for the purpose of this manuscript.

**Statistical reporting**

- Statistical analysis methods should be described and justified
- Raw data should be presented in figures whenever informative to do so (typically when N per group is less than 10)
- For each experiment, you should identify the statistical tests used, exact values of N, definitions of center, methods of multiple test correction, and dispersion and precision measures (e.g., mean, median, SD, SEM, confidence intervals; and, for the major substantive results, a measure of effect size (e.g., Pearson's r, Cohen's d)
- Report exact p-values wherever possible alongside the summary statistics and 95% confidence intervals. These should be reported for all key questions and not only when the p-value is less than 0.05.

Please outline where this information can be found within the submission (e.g., sections or figure legends), or explain why this information doesn’t apply to your submission:

A summary of the statistical analysis performed can be found in the legends for Fig. 2C, 3C, 5B, and 8C, the legends of supplements 7 and 9 of Fig. 1, and the legend of supplement 2 of Fig. 5. Further details regarding statistical analysis, including the statistical tests used, methods of correction and exact p-values can be found in the Materials and Methods section of the manuscript under the heading *Statistical analysis of experimental data*.

(For large datasets, or papers with a very large number of statistical tests, you may upload a single table file with tests, Ns, etc., with reference to sections in the manuscript.)

**Group allocation**

- Indicate how samples were allocated into experimental groups (in the case of clinical studies, please specify allocation to treatment method); if randomization was used, please also state if restricted randomization was applied
- Indicate if masking was used during group allocation, data collection and/or data analysis

Please outline where this information can be found within the submission (e.g., sections or figure legends), or explain why this information doesn’t apply to your submission:

The only experiment to which this is applicable is the *in vivo* survival assay presented in Fig. 8C. Information on sample allocation and masking is provided in the Materials and Methods under the heading *Galleria mellonella survival assay.*

**Additional data files (“source data”)**

- We encourage you to upload relevant additional data files, such as numerical data that are represented as a graph in a figure, or as a summary table
- Where provided, these should be in the most useful format, and they can be uploaded as “Source data” files linked to a main figure or table
- Include model definition files including the full list of parameters used
- Include code used for data analysis (e.g., R, MatLab)
- Avoid stating that data files are “available upon request”

Please indicate the figures or tables for which source data files have been provided:

“Source Data” files have been included in the form of Supplementary Table 2, a large format Excel table containing all MIC values recorded and used for the generation of Fig. 1B, Fig. 1 - figure supplement 2, and Fig. 5B. Supplementary Table 1 is a stand-alone item; it is a large format Excel table containing the output of the beta-lactamase bioinformatics analysis. Both these Excel files are provided separately, because they are too large to fit in the Supplementary Information file. Moreover, “Source Data” files have been provided for all figure panels containing immunoblots, namely Figures 2A, 2B, 3A, 4A, 4B,4C and 5C. Both the raw blots as well as images indicating the exact lanes used for the generation of the aforementioned figure panels have been provided.
